# Vineyard Pruning Extracts as Natural Antioxidants for Biodiesel Stability: Experimental Tests and Preliminary Life Cycle Assessment

**DOI:** 10.1021/acssuschemeng.3c00764

**Published:** 2023-05-17

**Authors:** Olena Dorosh, Elena Surra, Mário Eusebio, Ana L. Monteiro, Jorge C. Ribeiro, Nuno F. M. Branco, Manuela M. Moreira, Andreia F. Peixoto, Luís M. N. B. F. Santos, Cristina Delerue-Matos

**Affiliations:** † REQUIMTE/LAQV, Instituto Superior de Engenharia do Porto, Instituto Politécnico do Porto, rua Dr. António Bernardino de Almeida, 4249-015 Porto, Portugal; ‡ REQUIMTE/LAQV, Departamento de Química, Faculdade de Ciências e Tecnologia da Universidade Nova de Lisboa, Quinta da Torre, 2829-516 Caparica, Portugal; § Petrogal, S.A., Refinaria de Matosinhos, Rua Belchior Robles, 4451-852 Leça da Palmeira, Portugal; ∥ CIQUP, Institute of Molecular Sciences (IMS) - Departamento de Química e Bioquímica, Faculdade de Ciências da Universidade do Porto, Rua do Campo Alegre, P-4169-007 Porto, Portugal; ⊥ REQUIMTE/LAQV, Departamento de Química e Bioquímica, Faculdade de Ciências, Universidade do Porto, Rua do Campo Alegre s/n, 4169-007 Porto, Portugal

**Keywords:** vineyard pruning waste, environmental sustainability, subcritical water extraction; natural antioxidants, biodiesel, life cycle assessment

## Abstract

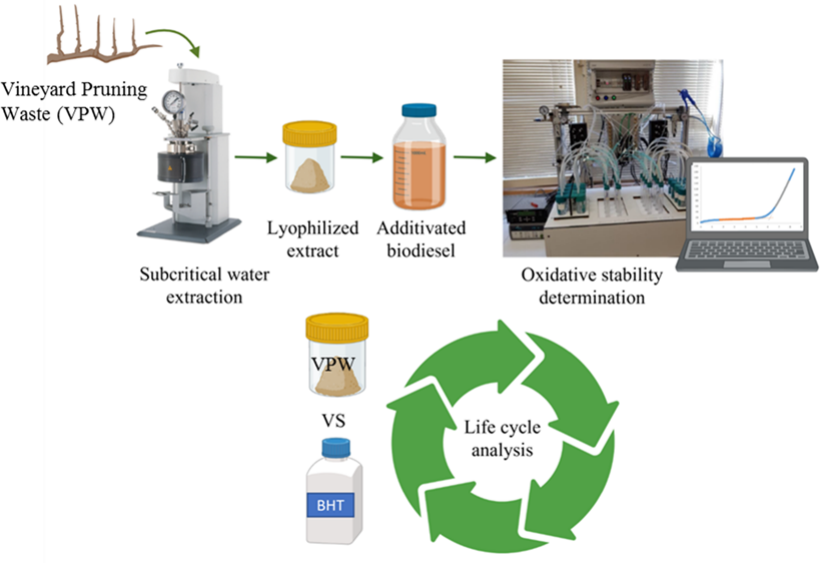

The control of the oxidative stability of biodiesel and blends of biodiesel with diesel is one of the major concerns of the biofuel industry. The oxidative degradation of biodiesel can be accelerated by several factors, and this is most critical in the so-called second generation biodiesel, which is produced from low-cost raw materials with lower environmental impacts. The addition of antioxidants is imperative to ensure the oxidative stability of biodiesel, and these are considered products of high commercial value. The antioxidants currently available on the market are from synthetic origin, so the existence/availability of alternative antioxidants of natural origin (less dependent on fossil sources) at a competitive price presents itself as a strong business opportunity. This work describes and characterizes a sustainable alternative to synthetic antioxidants used in the biodiesel market developed from extracts of vineyard pruning waste (VPW), which are naturally rich in phenolic compounds with antioxidant properties. A hydrothermal extraction process was applied as a more efficient and sustainable technology than the conventional one with the potential of the extracts as antioxidant additives in biodiesel evaluated in Rancitech equipment. The VPW extract showed comparable antioxidant activity as the commercial antioxidant butylated hydroxytoluene (BHT) typically used in biodiesel. The stability of the biodiesel is dependent from the amount of the extract added. Further, for the first time, the assessment of the environmental impacts of using natural extracts to control the oxidative stability of biodiesel in the production process is also discussed as a key factor of the process environmental sustainability.

## Abstract 2

Evaluation of the sustainability of using vineyard pruning waste extracts as natural additives for biodiesel production is discussed, along with comparative impacts with butylated hydroxytoluene.

## Introduction

Biodiesel is an environmental friendly alternative to fossil fuels for compression ignition (CI) engines, as it resembles fossil-derived diesel, provides less harmful emissions, and has the inherent advantages of being renewable and biodegradable.^1−3^ Biodiesel is composed of a mixture of fatty acid methyl (or ethyl) esters (FAME/FAEE) generally obtained from the transesterification reaction of vegetable oils, waste cooking oil, or animal fats with an alcohol (methanol or ethanol) in the presence of a catalyst.^4^ One of the main challenges for the commercial use of biodiesel is its higher tendency to oxidative degradation than fossil fuels.^5^ The biodiesel degree of instability depends on the fatty acid profile of the produced FAMEs/FAEEs, the number of intrinsic natural antioxidants, solubility, type of chemical structure, activation energy, and redox-reaction potentials, among others.^6^ These properties often determine the biodiesel strengths to resist storage conditions such as light, air, heat, and metal ions, since its degradation occurs through a series of classical free radical chain reactions and makes biodiesel storage for medium–long term use a challenge for the industrial and scientific community.^5,6^ The oxidation process occurs in three consecutive steps categorized as initiation, propagation, and termination. The initiation, step 1, can be triggered by thermal decomposition of the hydrocarbon RH or by its reaction with a chemical initiator. The propagation step occurs through reactions 2 and 3, forming carbonyl oxidation products. Finally, the termination step can occur through reactions 4 and 5 with the recombination of free radicals and the formation of stable products. The inhibition process can be done by using antioxidants to prevent the formation of free radicals by interrupting and decelerating the propagation propagation chain (eq 6), inhibiting the automatic oxidation of biodiesel and improving biodiesel resistance to oxidation.^7,8^
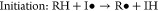
1
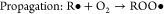
2
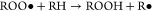
3
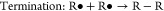
4
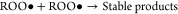
5
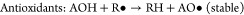
6

Currently and industrially, biodiesel oxidative stability is guaranteed exclusively by the addition of fossil-based antioxidants, as butylated hydroxyanisole (BHA), *tert*-butylhydroquinone (TBHQ), 2-ethylhexyl nitrate (EHN), butylated hydroxytoluene (BHT), or blends.^9^ These additives, in most of the cases, present acute toxicity for the aquatic environment^10,11^ and suspected carcinogenic effects^12^ for humans; thus, the development of new renewable and less harmful alternatives is mandatory.

Several agro-forestry wastes showed promising potential to be used as source of bioactive antioxidant compounds for biodiesel. Schaumlöffel et al.^13^ tested the efficiency of extracts from different lignocellulosic materials, namely, acacia (*Acacia mearnsii*), chestnut (*Castanea sativa*), and quebracho (*Schinopsis lorentzii*), as natural antioxidants for soybean biodiesel. The authors reported that 350 ppm of extract, mainly composed by tannins, mixed with 1.59 wt % of triethanolamine was more effective in enhancing the oxidative stability of soybean biodiesel than synthetic TBHQ.^13^ Devi et al.^14^ evaluated the effect of *Thuja oreantalis* L. leaf extract (obtained by leaf extraction with 100% of ethanol) on the oxidative stability of biodiesel produced from waste cooking oil through transesterification process. A total of six concentrations of extract were studied in order to evaluate their antioxidant activity in biodiesel observing that at least 100 ppm of extract allowed one to comply with the the requirements of the commercial biodiesel quality standard^15^ (6 h). Other authors also reported the increase of the oxidation induction period (IP) of biodiesel using different amounts of natural antioxidants obtained from curcumin, bilberry, oregano, and basil.^16,17^ Kumar et al. optimized the extraction experiments of *T. cordifolia* stems, varying solvent composition (methanol and water), extraction time, and extraction temperature.^18^ The extract was reasonably soluble in biodiesel, and extract concentrations higher than 600 ppm allowed extending the oxidation IP of biodiesel.

Recently, vineyard pruning waste (VPW) has had increasing attention among the scientific community since it is naturally rich in polyphenols that can be used as a source of bioactive compounds with high antioxidant potential.^19,20^ With an average yield of 1.75 t/ha, 1.3 × 10^4^ kt of VPW is produced in Europe per year, whereas in Portugal, with an extension of 174,000 ha of cultivated vineyards, 304 kt of VPW is annually available.^20,21^ Traditionally, it is commonly burned in the fields, causing high GHG emissions, or ground to be incorporated in the soil as an amendment.^22^

One of the parameters that mostly affects the amount of bioactive compounds recovered from VPW is the extraction technique.^19^ In fact, the same authors^23^ tested different techniques, conventional extraction, microwave-assisted extraction, and subcritical water extraction (SWE), and reported values of DPPH-RSA ranging from 8.31 to 35.3 mg Trolox equivalents/g dry weight demonstrating not only the high potential of VPW as an antioxidant precursor but also the importance to explore different extraction technologies to improve the extraction yield and to potentiate the commercialization of this natural antioxidant unexploited resource.

Among the different techniques that can be used for the extraction of antioxidant compounds from VPW,^19^ SWE was the one that stood out the most due to the high sustainability and easy operation.^23−25^ This technique is based only on the use of water as solvent and allows efficient extraction of the antioxidant compounds. These compounds can be efficiently incorporated in biodiesel after proper dissolution in a solvent to enhance the solubility parameters.^26,27^ Furthermore, the additions of oxygenated additives such as diethyl ether, ethanol, pentanol, decanol, ethylene glycol, propanol, and benzyl alcohol (aromatic alcohol) to biodiesel and biodiesel/diesel blends have been recently explored, and their impacts in engines evaluated. Most of the works showed promising results in terms of performance parameter improvements, like engine efficiency, emissions, and combustion, although the results depend on the amount of additive incorporated in biodiesel or blends.^28−31^

The prudent assessment of the environmental impacts during the development of the production process of VPW extracts as biodiesel additives is of the utmost importance as it allows one to embed sustainability considerations in its design improving the overall sustainability of the system.^32^ The environmental life cycle analysis (E-LCA) methodology is a highly consolidated and a widely used tool able to support the decision-making process of successive research pathways and subsequent future investments.^33^

The present work aims to (i) assess the suitability of VPW extracts as biodiesel antioxidants and (ii) provide a preliminary E-LCA analysis to compare the environmental benefits generated by the VPW extract production versus BHT, one of the most used fossil-based competitors.

This work is of unique importance for the future developments of natural antioxidants based on lignocellulosic extracts highlighting several advantages and weaknesses, on which future research steps must be preferentially focused.

## Materials and Methods

### Materials

VPW from the Touriga Nacional *Vitis vinifera* variety was randomly collected at Quinta dos Carvalhais owned by Sogrape Vinhos, S.A, and located at Mangualde, north of Portugal. The VPW samples were oven dried (Model no. 2000208, J. P. Selecta, Barcelona, Spain) at 40 °C for 24 h and milled (ZM200, Retsch, Porto, Portugal). The milled VPW was sieved to particle size lower than 1 mm and stored in polyethylene bags at room temperature until use.

Industrial biodiesel (antioxidant free) samples were supplied by Enerfuel S.A. (Portugal), produced by alkaline transesterification of recycled vegetable oils and animal fats in the presence of a catalyst (potassium methoxide) followed by a purification step by distillation^34^ and stored at room temperature until use. BHT was purchased from Sigma-Aldrich (≥99%), hexane (certified AR for analysis, 95% *n*-hexane approx) and propan-2-ol (certified AR for analysis) from Fisher-Chemical, and butanol (EMPLURA) and benzyl alcohol (EMPROVE) from Merck.

### Vineyard Pruning Extract Preparation

The VPW extract was obtained by submitting 20 g of milled VPW to SWE at 280 °C for 32 min in a Parr Series 4560 reactor connected to the Parr 4848 reactor controller, in the presence of 200 mL of water at 250 rpm.^35^ After cooling, the extract was centrifuged at 5000 rpm (Heraeus Megafuge 16 Centrifuge Series, Thermo Scientific, Waltham, MA, USA) for 15 min at 20 °C, chilled at −80 °C, lyophilized (Edwards lyophilizer) for 48 h, and stored at 4 °C until use.

### VPW Extract as Antioxidant for Biodiesel

To guarantee the VPW extract complete dissolution in the biodiesel, solubility tests of the VPW extract in different organic solvents were performed. The selection of the solvents was based on the green principle of avoiding the use of harmful organic solvents, on their capacity to solubilize the extract, and on their solubility in biodiesel.^27^ For this purpose, 200 mg of VPW extract was dissolved in 10 mL of hexane, butanol, propan-2-ol, benzyl alcohol, hexane:propan-2-ol 50:50 (v/v), butanol:benzyl alcohol 50:50 (v/v), and propan-2-ol:benzyl alcohol 50:50 (v/v), respectively. The solubility of VPW extracts in the selected solvents was assessed by visual analyses after dissolution at time 0 (*t*_0_) and after 24 h (*t*_24_) of storage in the dark. The formation of a dark solution without suspensions in the medium was assumed to be the visual indicator of the achievement of satisfactory solubility (Figure S1, Supporting Information).

### VPW Extract Characterization

The functional groups presented in the VPW extract were identified by the FTIR spectrum recorded as KBr pellets in a Jasco FT/IR Plus spectrophotometer. Scanning electron microscopy was carried out to obtain morphological features of the VPW extract using a high resolution (Schottky) environmental scanning electron microscope with X-ray microanalysis and electron backscattered diffraction analysis: FEI Quanta 400 FEG ESEM/EDAX Genesis X4M.

### Rancitech High Precision Oxidative Stability Apparatus

Rancitech is a high precision prototype apparatus, which performs 16 simultaneous and independent accelerated oxidative essays according to BS EN 14112:2020.^15^ Rancitech was designed, constructed, and tested previously at the laboratory of the Institute of Molecular Sciences (IMS), CIQUP, Chemistry and Biochemistry Department, Faculty of Science, University of Porto^7^ and includes two independent thermostable blocks with eight sample cavities each. The thermostable blocks, (two independent blocks, regions A1 and B1) are used to keep the samples at desired temperature (110 °C). The blocks were constructed of aluminum to achieve a good temperature homogeneity. Each block allocates eight sample cavities located symmetrically. A good heat power distribution was guaranteed using 10 heaters of 50 W each which are located symmetrically in each aluminum block. The temperature control with a resolution of ±0.1 °C was achieved using a high resolution PID controller (OMRON model E5DC) connected to a Pt100 (Class A) temperature sensor.

The stability and accuracy of the air flow is essential to achieve a high precision measurement of the oxidation stability (OS) induction time. The air flow regulation and control system of the purified air (produced in an oil free air compressor, RAVAGNANI model SD70/8) used a mass flow controller (BRONKHORST, model MV302).

Data acquisition and control system used a 6 digits data acquisition/data logger switch unit (Agilent model 34972, with a 22 channels multiplexer (34901A) and a 20 channel actuator/GP switch (34903A) boards). A customized software application developed in VEE-Pro 9.33 (from Keysight) “RANCI_Data” was developed to perform the data acquisition and control of the Rancitech apparatus.

In order to increase the accuracy and repeatability of the calculation of the oxidative stability time (IP), a customized software application “RANCI_Cal” was developed in VEE Pro. The OS time (IP) was derived from the interception of the two time/conductivity linear regions according to BS EN 14112:2020.^15^Figure S2 presents an overview of the typical results and the output of the RANCI_Data and RANCI_Cal software applications.

The results obtained in the Rancitech apparatus were compared with the results obtained in a commercial apparatus (METROHM, Rancimat model 743) using the same sample batch. The obtained results and detailed analyses are presented as Supporting Information (materials and methods and Figure S3) and are in excellent agreement and fulfill the repeatability interval needed to satisfy the BS EN 14112:2020.^15^ It was found that the obtained higher reproducibility and repeatability of the Rancitech apparatus when compared with the commercial model (METROHM, Rancimat model 743) is due to the improved air flow control and the better temperature control and homogeneity of the thermostable block.

### Accelerated Oxidative Tests

The VPW extract dissolved in the most efficient solvent as described in the VPW Extract as Antioxidant for Biodiesel section was added to 3 g of the same batch of raw biodiesel in concentrations of 600 ppm (BD-VPW_600_), 900 ppm (BD-VPW_900_), 1200 ppm (BD-VPW_1200_), and 1500 ppm (BD-VPW_1500_) and submitted to accelerated oxidative tests (measurement of IP). IP represents the duration (in hours/h) of the initial stage of the “rancidification” process before initiation of a drastically (fast) oxidation process for biodiesel storage stability assessment according to BS EN 14112:2020.^15^

In order to evaluate the aging effect on the antioxidant ability of the VPW extract, the IP was measured on BD-VPW_600_, BD-VPW_900_, BD-VPW_1200_, and BD-VPW_1500_ samples which were submitted at an accelerated aging process at 43 °C according to ASTM D4625-21.^36^ The IP of the preaged sample was measured right after the accelerated oxidative test (*t*_0_) and after 7 days (*t*_7_), 14 days (*t*_14_), 21 days (*t*_21_), 35 days (*t*_35_), 49 days (*t*_49_), and 63 days (*t*_63_) of accelerated aging stage.

The results obtained on preaged and aged biodiesel samples were compared to those obtained with biodiesel samples with 600 ppm of synthetic BHT additive (BD-BHT_600_) and BHT dissolved in benzyl alcohol (BD-BHT_600-BA_).^7^ It was found that 600 ppm is the concentration of BHT able to guarantee the minimum IP of 8 h required by BS EN 14112:2020^15^ for commercial biodiesel, previously submitted to the same accelerated oxidation tests.

Preaged and aged IPs of standalone biodiesel (BD) and biodiesel additivated with benzylic alcohol (BD-BA), both used as controls, were also measured.

Flash point tests were carried out using ISO 2719 – Procedure C (applicable to fatty acid methyl esters (FAME)).

### DPPH-Free Radical Scavenging Activity

The antioxidant activity of the standalone VPW extract in benzyl alcohol (1 mg/L) was assessed submitting it to the 2,2′-diphenyl-1-picrylhydrazyl radical scavenging activity (DPPH-RSA) test according to the methodology described by Dorosh et al.^25^ For this purpose, the absorbances of the preaged sample at time 0 (*t*_0_) and after 63 days of accelerated aging (*t*_63_) samples were measured at 515 nm using a Synergy HT microplate reader (BioTek Instruments, USA) equipped with the Gen5 2.00 program. The assays were performed in triplicate and the results expressed in milligrams of Trolox equivalents (TE) per gram of dry extract (mg TE/g de).

## Life Cycle Assessment

### Goal and Scope

Based on the laboratory results obtained in this work, which were properly adapted, upscaled, and integrated with literature data, an E-LCA was developed according to ISO 14040^32^ and 14044^33^ standards. This E-LCA aims for the comparison of the forecast environmental impacts, generated by the production of the VPW extracts, to the impacts associated with the production of the fossil-derived antioxidant BHT. To compare these two different products, 1 L of biodiesel was chosen as a functional unit (FU), since it is consistent with the goal of the study and with all functions of the systems.^32,33,37^

**Figure 1:**
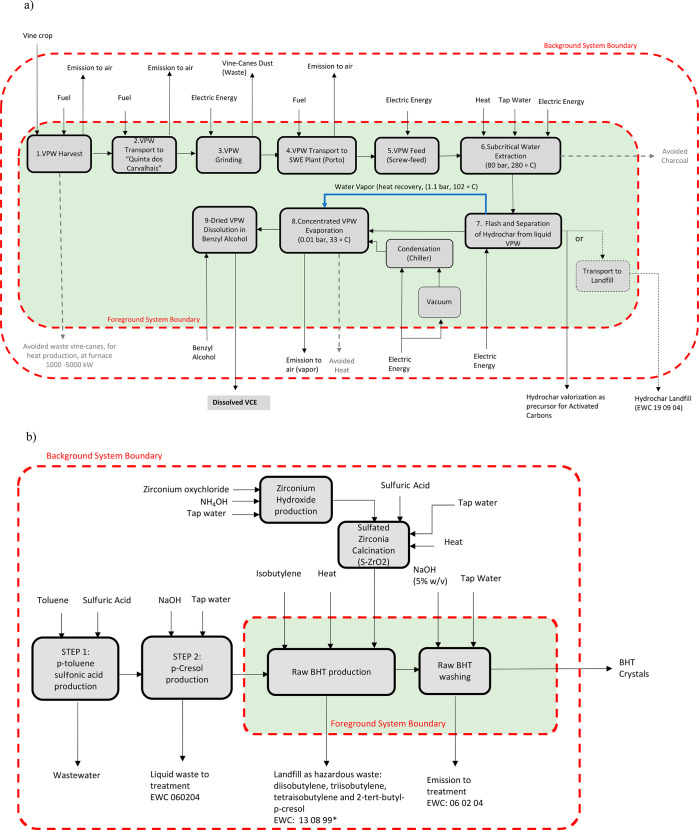
Flowsheets of the production for (a) VPW extract and (b) commercial BHT.

Figure 1a and b reports the flowsheet of the two systems studied and the corresponding foreground and background systems boundaries.^38^ The foreground system boundary refers to the additive production processes themselves, whereas the background system boundary includes the indirect processes involved in the production of the raw materials, energy, and processes not directly related with the production of the additive. For this study a “cradle-to-gate” approach is adopted.

Figure 1a reports the hypothesized VPW extract’s production process: it includes the implementation of a SWE plant in the peri urban area of the city of Porto, with the capacity of processing 13.4 t/y of VPW, which is the biomass amount that can potentially be supplied by the “Quinta dos Carvalhais”, owned by Sogrape S.A., located in Mangualde, morth of Portugal, at approximately 150 km from the city of Porto, which, according to the results obtained in this work, lately described, can satisfy 2.5% of the total amount of biodiesel antioxidant required for the Portuguese market in 2018.^39^ The employed SWE conditions also produced a hydrochar (solid) fraction that can be used as catalyst or catalyst support^40^ and precursor for activated carbons (AC), among other applications, contributing to the integrated valorization of VPW. In this work, the environmental impacts associated with production of VPW extract were analyzed considering (i) the valorization of the produced hydrochars as precursor for AC and (ii) direct hydrochar landfill (Figure 1a).

The production process of the fossil-derived BHT considered in the present work is reported in Figure 1b. BHT production is based on the alkylation of *p*-cresol with gaseous isobutylene in the presence of a catalyst.

For the allocation of the impacts, it was chosen to use the consequential approach, which allows estimating how and how much the production of the developed VPW extract affects the global environmental burdens of the system studied. This approach describes how environmentally relevant flows are affected by possible decisions.^41,42^

Accordingly, the allocation of the impacts used in this work follows the system expansion methodology proposed by Clift et al.,^43^ which consists in the identification of the product that can replace a less sustainable product already present in the market. This approach is known as the “avoided-burden method”. In the present study, these products were related with the (i) valorization of VPW, (ii) valorization of hydrochar, and (iii) energy integration in the plant design. This LCA study was developed by using the ReCiPe2016(H) methodology of calculation, which converts, through proper characterization factors, the elementary flows of the inputs into 18 environmental indicators at the midpoint level and three at the endpoint level.^44^ The midpoint level focuses on single environmental issues (i.e., global warming, human carcinogenic and noncarcinogenic toxicity, etc.). The endpoint level is directly related to the damage caused by the induced impacts on the three areas of protection, namely, (i) human health, expressed in disease adjusted life year (DALY), which represents the loss of the equivalent of one year of full health,^45^ (ii) ecosystems, measured in terms of number of potentially disappeared species (species.y), and (iii) resources, which is assessed as increased costs for extracting 1 kg of *i* resource and is linked with the resource availability (USD2013).^46^ The perspective adopted was the “Hierarquist” (H) model, which assumes that future damages can be avoided if proper management or future technology will be effective as a function of time and human expectations. The software package used for this study was the SimaPro Version 9.1.1.7 from pre-Sustainability (Le Amersfoort, The Nederlands) run in Windows 10 and equipped with the Ecoinvent 3.7 database.

### Life Cycle Inventory

The VPW extract production process was divided in nine subprocesses as reported in Figure 1a. Subprocesses 1–5 were modeled in batch, whereas subprocesses 6–9 were designed continuously to allow energy integration and environmental impacs reduction. The subprocesses 6–9 were modeled using the AspenOne AspenBaciEngineering software package, by Aspentech.^47^ In detail, the following subprocesses were considered: (1) VPW harvest, (2)VPW transport to “Quinta dos Carvalhais”, (3)VPW grinding, (4) dried VPW transport to SWE plant (Porto, Portugal), (5) VPW feed (screw-feed), (6) subcritical water extraction, (7) flash and separation of hydrochar from liquid VPW extract, (8) concentrated VPW extract evaporation, and (9) dried VPW extract dissolution in benzyl alcohol. The detailed description of the assumptions made for the development of the subprocesses 1–9 as well as the engineering choices and their scale-up design are reported in Table S1 (Supporting Information), and the life cycle inventories are reported in Tables S2–S5 (Supporting Information).

**Figure 2:**
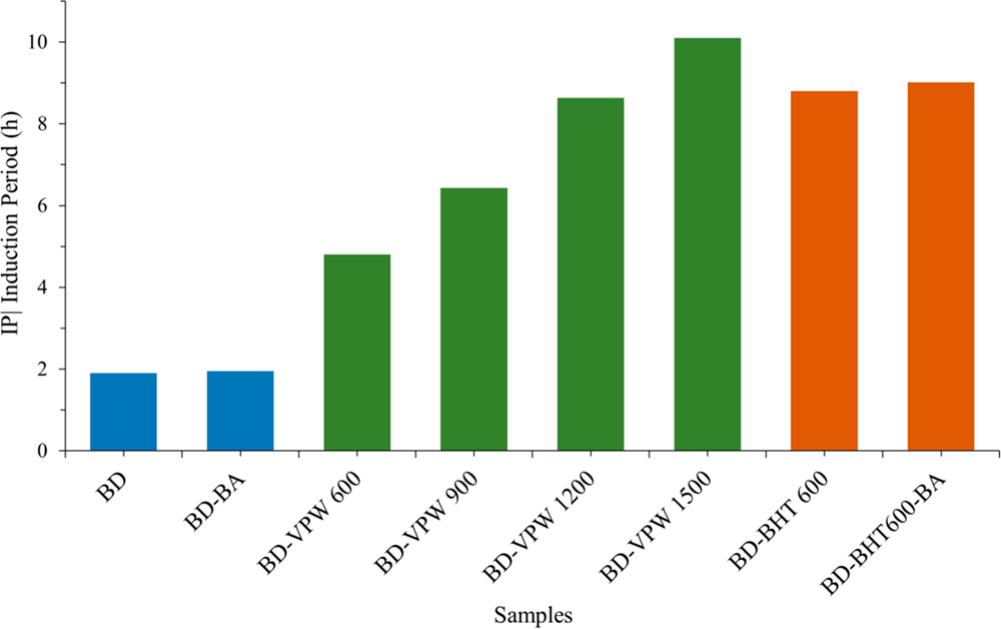
Mean induction period (IP) of preaged samples (*t*_0_). The IP overall uncertainty, *U*(h), is estimated as *U*(h) = ±(0.02 × IP + 0.1). BD: biodiesel (antioxidant free); BD-BA: biodiesel with benzylic alcohol (3 mL); BD-VPW600:600 ppm (BD-VPW_600_); BD-VPW900:900 ppm (BD-VPW_900_); BD-VPW1200:1200 ppm (BD-VPW_1200_): BD-VPW1500:1500 ppm (BD-VPW_1500_); BD-BHT600: BHT additive (BD-BHT_600_); BD-BHT600-BA: BHT dissolved in benzyl alcohol (BD-BHT_600-BA_).

The description of the assumption made for the development of the subprocesses considered for modeling the BHT production process reported in Figure 2b, as well as its life cycle inventory, are reported in Tables S6 and S7 (Supporting Information), respectively.

## Results and Discussion

The chemical composition (fatty acid methyl ester) and some properties of the biodiesel (antioxidant free) sample were performed by Petrogal, S.A., Refinaria de Matosinhos and is available in Tables S8 and S9 in the Supporting Information. The VPW extract was characterized by FTIR and SEM, and the respective spectrum and images are presented in Figure S4 of Supporting Information. The FTIR spectrum of the complex mixture showed the presence of typical functional groups being the most significant with the presence of bands at 3400 and 2900 cm^–1^ attributed to −OH stretching vibrations confirming the presence of OH and carboxylic acid groups, which can be assigned to the Maillard reaction products. The presence of aldehydes alkenes, aromatics, ethers, primer alcohols, and phenols can also be detected, as observed by other authors in the FTIR analysis of a ginger extract^48^ and by the presence of typical FTIR bands (Figure S4a). The external morphology of the VPW extract was studied by SEM, as shown in Figure S4b). The scanning electron micrograph of the VPW extract surface presents as expected an irregular morphology, with the presence of C and O as major elements observed by EDS analysis.

Based on the visual observation of the tested solvents, benzyl alcohol demonstrated to be the one which provided the highest solubility of the VPW extract since it allows the generation of a solution free of significant deposits at *t*_0_ and mostly after 24 h of dark storage (*t*_24_). Benzyl alcohol was chosen as the candidate for enhancing the VPW solubilization in biodiesel in the subsequent experiments and for LCA modeling. Indeed, benzyl alcohol represents an attractive and safe choice due to its low volatility and relatively low toxicity. Moreover, benzyl alcohol is a very accessible product, since in 2020, it was the 3674th most traded product in the world, with a total trade of 172 M US$, representing 0.001% of the total word trade.^49^ Industrial production of benzyl alcohol is traditionally made by hydrolysis of benzyl chloride or hydrogenation of benzaldehyde.^50^

Figure 2 reports the results of the accelerated oxidative test of preaged samples (t_0_) obtained in the Rancitech apparatus according to the BS EN 14112:2020.

The addition of VPW extract to biodiesel increased linearly the biodiesel oxidative stability (and consequently the IP), providing values of IP ranging from 4.8 h (BD-VPW_600_) to 10.1 h (BD-VPW_1500_) (Figure 2). It must be noticed that only the BD-VPW_1200_ and BD-VPW_1500_ samples comply with the BS EN 14112:2020 requirements, showing an IP of 8.6 and 10.1 h, respectively. Moreover, BD-VPW_1500_ provided higher oxidative stability than biodiesel additivated with the synthetic BHT either in the presence (BD-BHT_600-BA_) or absence of benzyl alcohol (BD-BHT_600_), demonstrating the high potential of this novel natural antioxidant as an alternative to fossil-based commercial antioxidants.

Benzyl alcohol did not interfere in the biodiesel oxidative stability, since no significant differences were observed in the IP of standalone biodiesel (BD) and biodiesel mixed with benzyl alcohol (BD-BA), as well as in the IP of biodiesel additivated with BHT (BD- BHT_600_) and with BHT dissolved in benzyl alcohol (BD-BHT_600-BA_) (Figure 2).

**Figure 3:**
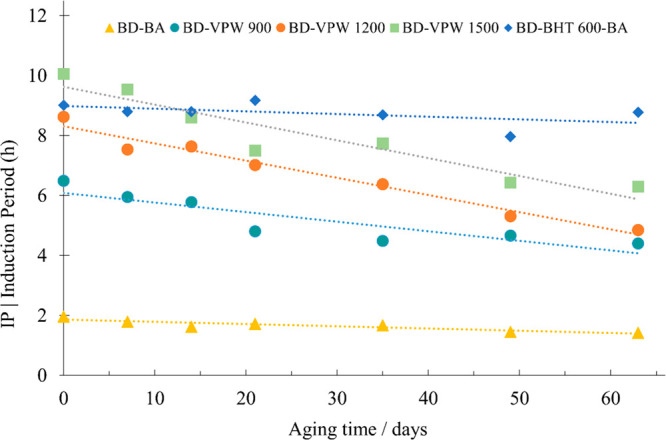
Evolution of the oxidation stability induction period (IP) along the aging time (days) at 43 °C. The IP overall uncertainty, *U*(h), is estimated as *U* (h) = ±(0.02 × IP + 0.1).

Figure 3 presents the results of the change of the IP from 0 (*t*_0_) to 63 days (*t*_63_) of accelerated aging time describing the behavior of the biodiesel samples during storage. In the methodology used to perform the accelerated aging study, 1 week of storage at 43 °C corresponds to 4 weeks at 21 °C (underground, ambient storage).^51^ It was observed that the decrease in the oxidative stability (IP) of all biodiesel samples additivated with VPW is related with the initial concentration of the VPW, which can be related with a partial thermal decomposition of some active compounds present in the VPW extract. Due to the lower stability and complexity of the natural antioxidants, it is expected that this could have an anomalous behavior on the accelerated aging process resulting in wrong/unexpected results for the aging time prediction ratio (1 to 4) from the accelerated methodology, and for that reason, the analysis of the aging data should be taken carefully in natural antioxidants. It was found that the minimum 8 h IP required by BS EN 14112:2020 is guaranteed for a maximum of 4 days (corresponding to 16 days at 21 °C) by BD-BHT_1200_ and for 32 days (corresponding to 128 days at 21 °C) by BD-BHT_1500_. This means that the BD-BHT_1500_ samples can be stable during approximately 120 days of storage underground at 21 °C. These results are in agreement with previous findings obtained by de Sousa et al.^16,17^ and Devi et al.^14^ The first authors^15,17^ reported that the IP of biodiesel with 1500 ppm of curcumin decreased from 9.1 h to approximately 6.5 h after 180 days of storage in the dark at 25 ± 0.5 °C.^14^ While Devi et al.^14^ observed that the IP of samples containing 1000 and 2000 ppm of *Thuja oreantalis* L. leaf extract decreased from 9.62 and 11.04 h to 8.0 and 9.2 h, after 90 days of storage in the dark at 25 ± 0.5 °C.

The results obtained in this work confirm that higher concentrations of VPW extract increase the oxidative stability not only for fresh but also for aged biodiesel samples, suggesting that it is possible to improve the biodiesel long-term storage stability by improving the VPW extract concentration in biodiesel samples. These results suggest that this biodiesel stabily is related to the phenolic antioxidants present in the VPW extract which can react with lipid radicals, blocking free radical reactions and destroying the growth of free radical chains. According to Chen et al.,^8^ the antioxidant mechanism against biodiesel promoted by a phenolic reach extract acts in order to terminate the formation of free radicals (R), first and second by antioxidant absortion of the free radicals. The antioxidant reacts with free radical R• and reduces the free radical R to the original RH (eq 6).

Nevertheless, the results of this aging study must be interpreted with caution. It should be noted that oxygen exposure, contamination from metals and other radical initiators, water exposure, light exposure, and heat could all contribute to the degradation of fuel quality.^52^ The conditions used for aging samples in this experiment are clean, well-maintained, dark, quiescent storage and do not mimic necessarily the real conditions applied to biodiesel/blends storage situations, indicating that long-term storage of biodiesel is possible under clean and controllable conditions. Induction time decrease indicated loss of stability (consumption of antioxidant) prior to biodiesel degradation; therefore, induction time monitoring is recommended for predicting quality changes during storage.

Finally, BD-BHT_600_ showed an approximately constant IP of 8 h for 63 days, which could be related with high thermal stability of the BD-BHT (Figure 3).

It must be noticed that BD-BA provided very low IP, with an approximately constant value of 1.9 h during the 63 days of storage indicating that the oxidative stability of biodiesel itself in absence of a specific antioxidant additive is not directly affected by the duration of storage under the studied conditions. Other authors demonstrated that the addition of benzyl alcohol to diesel or blends showed promising results in terms of performance parameters like efficiency, emission, and combustion improvement.^30,53^ Flash point is a critical point to final biodiesel and diesel blends; in order to demonstrate that the addition of the VPW extract and benzyl alcohol additives does not affect the biodiesel flash point, the samples BD, BD-BA, BD-BHT 600, and BD-VPW 1500 were analyzed and the flash point measurements performed according to ISO 2719, Procedure C. The results are presented in Table S10 and show that the addition of BA or BHT to biodiesel contributed to a flash point reduction of ∼20% (from 158.0 °C to 126 and 128 °C, respectively). Nevertheless, for the BD-VPW 1500 sample (biodiesel + VPW dissolved in BA) a slight decreasing was observed (to 134 °C). All the obtained values meet the specified requirement limits in accordance with the 2019 EN 14214 standard, in which the flash point must be >120 °C to ensure performance and safety to the engines.

The DPPH-RSA assays on preaged *t*_0_ and aged *t*_63_ VPW extracts were also performed, and the results showed that antioxidant activity of the VPW extract decreased from 214 mgTE/g dw (*t*_0_) to 125 mgTE/g dw after aging (*t*_63_). This DPPH-RSA value decrease after accelerated aging suggests that some degradation of thermolabile compounds occurred, and consequently, the antioxidant activity of the VPW extracts in biodiesel has been affected for a long time.^54^ In previous works, Moreira et al.^23^ compared the antioxidant activity, using a DPPH-RSA assay, of *Tinta Roriz* (the variety source of VPW extract of this study) and *Touriga Nacional* from the Dão region, obtained from subcritical extraction methodology and found that the antioxidant activity of TN (9.5 ± 0.7 mgTE/g dw) was lower than that obtained for TR (15.2 ± 1.2 mgTE/g dw). Since these VPW varieties presented identical climatic and geographical factors, viticultural characteristics, and cultivation techniques, these results can be mostly attributed to the effect of the different variety. More recently, the same authors^24^ evaluated the antioxidant properties of different VPW subcritical water extracts as active ingredients in the cosmetic industry. The authors used six vineyard pruning varieties, namely, *Touriga Nacional* and *Tinta Roriz* from the Dão and Douro region and *Alvarinho* and *Loureiro* from the Minho region, concluding that the *Loureiro* variety has the highest DPPH-RSA (17.89 ± 093 mgTE/g dw). This study clear demonstrates that the VPW variety exerts a significant influence in the extraction efficiency and consequently on the extract’s antioxidant activity. Further, these authors also report that different environmental and microclimatic conditions could also be mainly responsible for the observed diferences. As the antioxidant activity of vineyard pruning extracts is directly correlated with the biodiesel stability, it could be interesting to use other variety extracts, as *Loureiro*, in order to evaluate their potential to inhibit the oxidative stability of biodiesel.

Based on the results obtained, BD-VPW_1500_ provided the highest oxidative stability than the other VPW extract concentrations tested, being able to accomplish the BS EN 14112:2020 requirements both on preaged and aged samples; thus, it is chosen as the candidate for LCA modeling.

### Life Cycle Assessment

Table 1 reports the forecast for midpoint environmental impacts associated with the production of VPW extracts hypothesizing the processing of all the vineyard pruning harvested at “Quinta dos Carvalhais”, taken as reference.

**Table 1:**
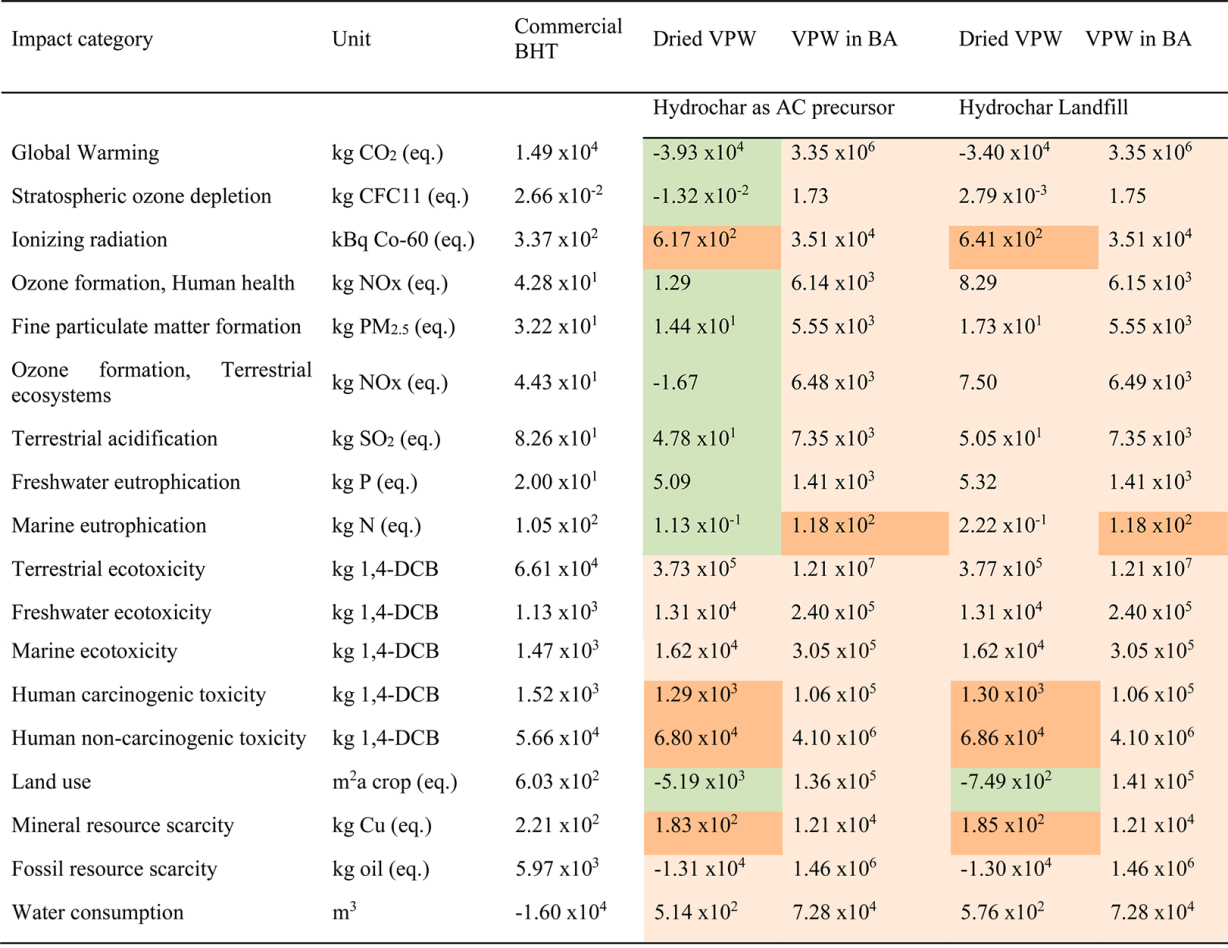
Total Impacts Calculated for Different VPW Scenarios Compared with Commercial BHT According to ReCiPe Midpoint (H) Method^a^

a Light orange and green cells represent the environmental impacts higher and lower than BHT (base-case scenario), respectively. Orange cells indicate the values which remain in the same order of magnitude as the BHT scenario. BA: benzyl alcohol; 1,4-DCB: diclorobenzene.

The production of dried VPW extract is environmental more sustainable (second and forth columns) than commercial BHT (first column) in most of the impact categories analyzed. However, when dried VPW extract is mixed with benzyl alcohol (third and fifth columns), the VPW extract produced cannot environmentally compete with the commercial fossil-based BHT, regardless if the hydrochar is valorized as a precursor for AC (third column) or landfilled (fifth column). These results are explained by the need of benzyl alcohol to dissolve VPW extract (to be further added to biodiesel), whose production process brings high indirect environmental loads since it is based on fossil toluene conversion.

Up to step 8 (Figure 1a), thanks to the energy integration approach used in the design of the upscaled process, the heat recovered from hot water vapor obtained during the flash of the SWE reactor (step 7, Figure 1a) and the avoided production of the equivalent amount of heat generated from natural gas turn the production of dried VPW extract environmentally more advantageous than commercial BHT in most of the categories of impact (second and third columns, Table 1).

Observing the scenarios for the dried VPW extract, negative impacts on the categories of global warming, fossil resource scarcity, and land use either if hydrochar is landfilled or valorized as a precursor of AC are obtained, whereas stratospheric ozone depletion, ozone formation, and terrestrial ecosystems categories provide negative impacts but only when hydrochar is valorized as a precursor for AC. This demonstrates that the avoided heat production obtained by process energy integration and avoided wood waste production due to vineyard pruning valorization (Tables S1 and S2, Supporting Information) overcome the impact associated with the dried VPW production process, providing environmental credits to the system in the mentioned categories. Moreover, the valorization of the produced hydrochar as an AC precursor^36,55^ is also more environmentally advantageous than its landfill, allowing lower environmental impacts in 16 of the 18 categories of impact analyzed for the dried VPW, specifically with percentages of reduction ranging from 1% (human carcinogenic and noncarcinogenic toxicity) to 549% (ozone formation, terrestrial ecosystems). Finally, dried VPW showed 1 order of magnitude higher environmental loads than commercial BHT in freshwater, marine, and terrestrial ecotoxicities, as well as in water consumption categories of impact. The higher values observed in freshwater and marine ecotoxicities of dried VPW are affected by the copper production process associated with the construction and distribution of an electric energy network. In fact, these indicators are affected, on average, by 37.7% by the treatment of scrap of copper by municipal incineration and by 16.9% of sulfidic tailings from the copper mine operation. The value of the terrestrial ecotoxicity impact of 53% is due to the basic copper production process at the mine. The higher impacts observed for the dried VPW scenarios for the water consumption category of impact are intrinsically associated with the SWE process, which is a water-based process; thus, associated impacts to water resource depletion are expected.

Table 2 reports the aggregated results of the impact assessment calculated at the endpoint (H) level and interpreted from a damage assessment perspective. These results define the damages caused by the impacts generated by the process analyzed in the three areas of protection: human health (DALY), ecosystems (species.y) and resources (USD2013).

**Table 2:**
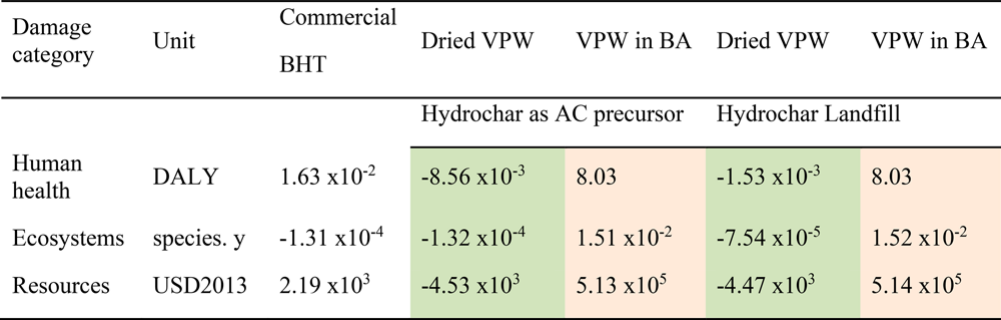
Total Impacts Aggregated in Human Health (DALY), Ecosystems (species.y), and Resources (USD2013) Areas of Protection Calculated According to ReCiPe Endpoint (H) Method^a^

a Light orange and green cells represent the environmental impacts higher and lower than BHT (base-case scenario), respectively.

As previously observed at the midpoint level in the 18 categories of impact analyzed (Table 1), the addition of benzyl alcohol to solubilize the dried VPW extract into biodiesel increases the impacts in the three areas of protection at least in 2 orders of magnitude, potentially representing the main reason responsible for the higher damages on human health, ecosystems, and resources categories. In the presence of benzyl alcohol, the hydrochar valorization as AC precursor production allows a slight reduction of the associated damages in the ecosystems and resources areas of protection.

Referring to dried VPW extract, before dissolution in benzyl alcohol, the valorization of hydrochar as an AC precursor can reduce the environmental loads associated with human health, ecosystems, and resources areas of protection by 153%, 0.15%, and 307%, when compared to commercial BHT. On the other hand, the landfill of the produced hydrochar brings the highest environmental loads in the ecosystems area of protection than commercial BHT, whereas human health and resources areas of protection continue to be more advantageous than BHT.

**Figure 4:**
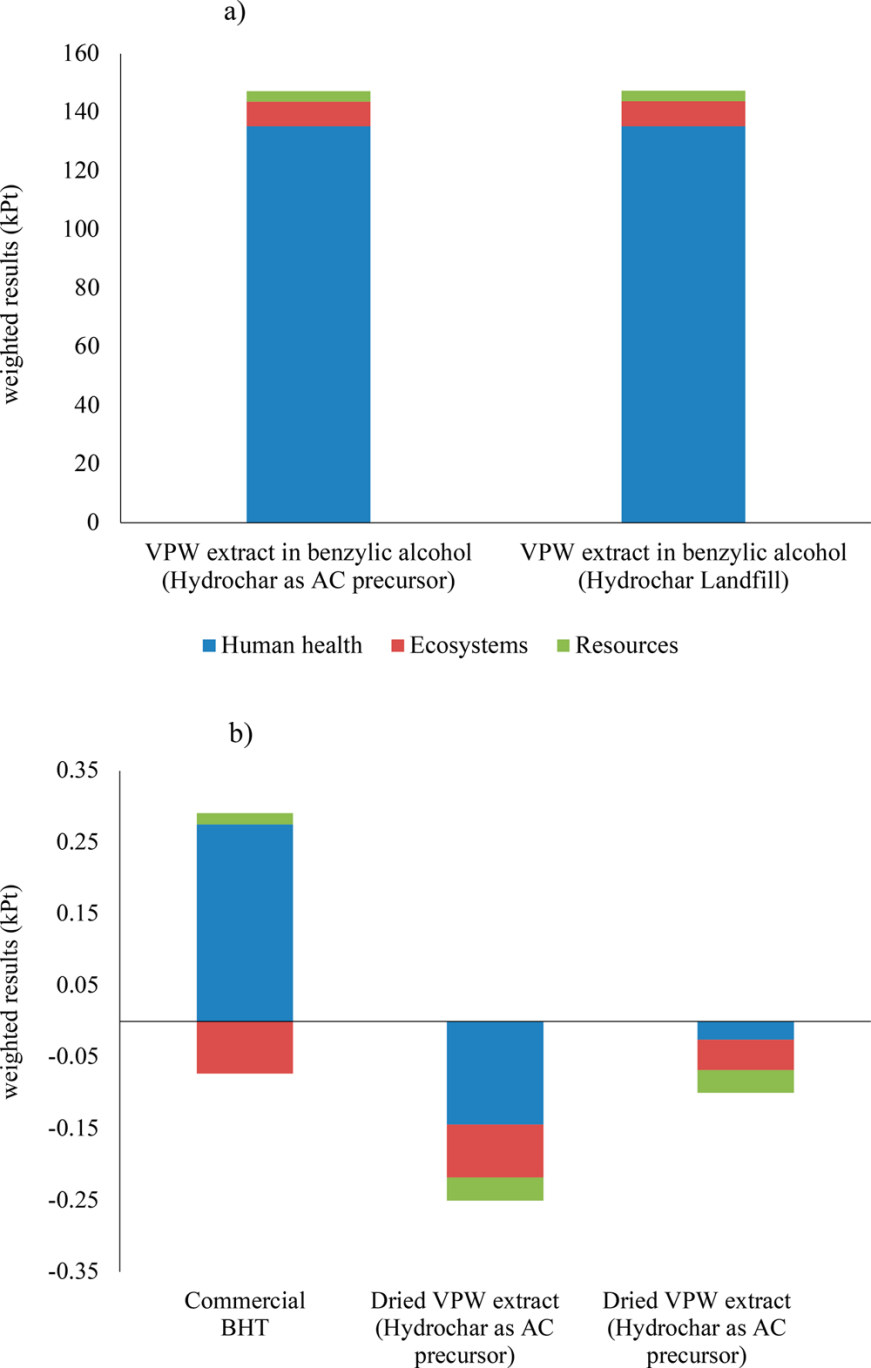
Total impacts weighted according to ReCiPe Endpoint (H) method and aggregated (kPt) in the three areas of protection: human health (blue), ecosystems (red), and resources (green): (a) scenarios for VPW extract dissolved in benzyl alcohol and (b) scenarios for commercial BHT and dried VPW extracts without solubilization in benzyl alcohol.

Figure 4a and b shows the total impacts compared through the “weighting” function. The “weighting” function allows comparing all the categories of impact through a single score (Pt) able to rank the impacts according to the importance of the effects they are able to trigger.^56^ The use of the “weighting” function means applying a value judgment to LCA results using the weighting factors included in the Recipe2016(H) method, which are elaborated according to the literature^57^ on four basic categories: distance from policies and scientific targets, monetization, and panel weighting. The “weighting” function can be very effective to integrate the results obtained at the endpoint in a strict damage assessment approach (Table 2) to easily communicate and compare the preliminary results for future decision making.

The information retrieved after the “weighting” function must be carefully interpreted, as it can be intrinsically controversial due the choice of the characterization factor used by the method used (ReCiPe2016) and also very useful in an early stage to define future research options, which is the case of the present work.

Figure 4a shows that the area of protection highly affected by the final VPW extract dissolved in benzyl alcohol is human health accounting, on average, for 92% of the final value, whereas resources and ecosystems contribute 2% and 6%, respectively. Analyzing the process contribution reported in Table S11 (Supporting Information) to the area of protection of human health is affected by a percentage higher than 14% by toluene production process, which is the raw material for benzyl alcohol production.

The highest environmental credits are achieved in the human health area of protection for the dried VPW scenario when hydrochar is valorized as an AC precursor (Figure 4b). This result is due to the contribution given by the avoided heat production provided by the recovery of the enthalpy of the hot water vapor stream obtained during the flash of the SWE reactor (step 7, Figure 1a and Tables S1–S2, Supporting Information). This heat recovery reduces the impact values of global warming and fine particulate matter formation, whereas the benefits obtained in stratospheric ozone depletion and ozone formation categories are mainly due to the avoided production of waste wood and its consequent incineration and, only secondarily, by the avoided heat production due to the energy integration design.

Regarding human carcinogenic and noncarcinogenic toxicities, the avoided treatment of spoil from lignite mining, as well as treatment of sulfidic tailings from coal mines, represent the highest contribution to the reduction of damages on human health. These processes are linked to the avoided production of fossil-based charcoal, lately used as an AC precursor.

Regarding the ecosystems, the environmental credits provided are quite similar by percentage for commercial BHT and dried VPW extract and are due to avoided wastewater production and heat recovery, respectively.

The results obtained by this preliminary E-LCA are of unique importance since they alert researchers about the high potential of the new natural antioxidant extract developed or warn about the key limitation associated with the process developed as the type and the amount of the solvent used to solubilize the extract in biodiesel can significantly affect its sustainability. The meticulous simulation developed set an important milestone for future research steps, highlighting its strengths and weaknesses.

### Sensitivity Analysis

Since the type and the amount of solvent demonstrated to be key factors for the overall sustainability of the system, due to the indirect impacts associated with nonrenewable raw materials, presence of metal catalysts, high temperatures, high pressures, and production of harmful byproducts during benzylic alcohol production process, a sensitivity analysis was performed. Namely, based on late promising solubility tests perfomed by the authors and the potential of obtained benzyl alcohol from biomass resources, Sensitivity Analysis-1 was conducted to assess the variation of the environmental impacts assuming the use of pentanol and ethylene glycol as alternative solvents to benzyl alcohol, which due to their physicochemical properties can be considered suitable to be blended in biodiesel with no negative effects on biofuel performances.^30^ For the comparison of these alternative solvents, the same VPW:solvent ratio (20 mgVPW/mLsolvent) was used as for benzyl alcohol. The scenarios analyzed were denominated “VPW in pentanol” and “VPW in ethylene glycol”, respectively.

Moreover, the changes of the environmental impacts by reducing 20% and 50% of the initial amount of the benzyl alcohol were calculated, using a VPW:BA ratio of 25 and of 40 mg_VPW_/mL_BA_ (Sensitivity Analysis-2). These latter scenarios were denominated “VPW in BA 25” and “VPW in BA 40”, respectively. For both Sensitivity Analysis-1 and -2, the followed methodology was the same for the previous analysis, and the most favorable scenario of hydrochar valorized as a precursor for AC was taken as reference. The results were compared with the previous results obtained for standalone benzyl alcohol and for commercial BHT already reported in Tables 1 and 2 (fifth column), and Tables S12 and S13 report the LCI of the scenarios studied in Sensitivity Analysis-1 and -2.

Tables S14 and S15 highlight that reducing the amount of benzyl alcohol up to half of the initial amount (“VPW in BA 40”) allows a decrease of the environmental impacts lower than 6% in 18 categories of impact analyzed both at midpoint and endpoint levels. For Sensitivity Analysis-1, the substitution of benzyl alcohol by ethylene glycol is able to reduce on average the environmental impacts by 48% at midpoint and endpoint levels when compared with “VPW in BA”, pointing out that ethylene glycol can be considered a promising alternative to benzyl alcohol. Future tests with reduced quantities of ethylene glycol and subsequent analysis of its compatibility in biodiesel/blends and therefore in engines can open new opportunities for future research steps. To date, none of the alternative scenarios studied in this work are able to overcame the environmental performance provided by fossil-based BHT (Tables S14 and S15).

## Conclusions

Biodiesel is prone to oxidation depending on the process production, feedstocks used, especially those from low-grade sources, and storage conditions, among others. The addition of natural extracts obtained from biomass rich in polyphenols represents a promising alternative to synthetic additives (fossil fuel derivatives). This work demonstrated the suitability of VPW extracts obtained by SWE as an antioxidant additive to promote the oxidative stability of biodiesel. The FTIR spectrum of the VPW extract complex mixture showed the presence of typical functional groups (e.g., carboxylic acids, aldehydes, and aromatics). The results obtained during the Rancitech-accelerated oxidative tests showed that the oxidative stabilities of the preaged and aged biodiesel samples are proportional to the VPW extract concentration added and that 1500 ppm of VPW extract (BD-VPW_1500_) allowed one to comply to the standard required by BS EN 14112:2020 for commercial biodiesel up to 32 days, which corresponds to 120 days storage at underground conditions at 21 °C. The slight decrease of the oxidative stability observed along time can be attributed to some VPW extract’s degradation reactions during the storage as confirmed by the results of the DPPH-RSA assays performed on standalone VPW extract samples. To overcome this limitation, the monitoring of the induction time of the biodiesel additivated by VPW extracts is crucial, and in case of decay, small amounts of VPW extract can be added to maintain the IP stable in the long term. Benzyl alcohol demonstrated to be an efficient solvent in both preaged and aged samples. The addition of VPW and benzyl alcohol does not allow a remarkable effect in the biodiesel flash point.

The assessment of the environmental impacts associated with the development of this novel VPW-based additive, already in an early stage of its technological development, helped to answer to two fundamental questions: (1) Is the developed production process of VPW extracts more environmentally sustainable when compared with the commercial synthetic BHT production? (2) Is the valorization of the hydrochar, produced as a byproduct during VPW extraction, as an AC precursor more sustainable than a landfill?

The results of the E-LCA clearly show that VPW extract is environmentally competitive with BHT up to its drying, also thanks to the energy integration step included in the design of the process of VPW extraction. However, the needs of benzyl alcohol to allow VPW extract incorporation into biodiesel cut off the environmental benefits of VPW extract when compared to fossil-based BHT. Nevertheless, the valorization of produced hydrochar as an AC precursor allows one to obtain additional environmental credits, which benefits the system, and is environmentaly highly recommended in line with a biorefinery concept. The results of the sensitivity analysis suggest that considering other solvents, such as ethylene glycol, or the reduction of the amount of benzyl alcohol can be considered the right approach for future investigations.

The results obtained provide crucial information about the choices made during the experimental work in terms of sustainability comparing with conventional solutions in the market. This preliminary simulation provides the fundamental information to achieve a future sustainable solution.

## Supporting Information Available

Detail description about materials and methods: Images of the sample preparation (Figure S1), information of RANCI_Data (right) and RANCI_Cal (left) software applications (Figure S2), comparation of results of the Rancitech with Metrohm apparatus Rancimat model 743 (Figure S3). Detail description about results and discussion: Information about VPW extract characterization (Figure S4), information about life cycle inventory assumptions and data (Tables S1–S7), information about biodiesel, chemical composition (Table S8), some other properties (Table S9), flash point (Table S10), information about process contributions to areas of protection of human health at endpoint (H) level (weighting) of VPW extract dissolved in benzylic alcohol (Table S11), inventory data of Sensitivity Analysis-1 and -2 (Tables S12 and S13), total impacts calculated for different scenarios of Sensitivity Analysis-1 and -2 (Table S14), and total impacts aggregated in human health (DALY), ecosystems (species.y), and resources (US$2013) areas of protection (Table S15). (PDF)
