# Supplementary material for: Vineyard Pruning Extracts as Natural Antioxidants for Biodiesel Stability: Experimental Tests and Preliminary Life Cycle Assessment
Source: ACS Sustain Chem Eng. 2023 May 17;11(21):8084–95. doi: 10.1021/acssuschemeng.3c00764 (PMC10230498; doi:10.1021/acssuschemeng.3c00764)
Supplement: Supplementary file 1 — sc3c00764_si_001.pdf [file sc3c00764_si_001.pdf]

Supporting information:

Vineyard pruning extracts as natural antioxidants for biodiesel stability: Experimental Tests and Preliminary Life Cycle Assessment

Olena Dorosh[1], Elena Surra[1], Mário Eusebio[2], Ana L. Monteiro[3], Jorge C. Ribeiro[3], Nuno F. M. Branco[4], Manuela M. Moreira*[1], Andreia F. Peixoto*[5], Luís M. N. B. F. Santos[4], Cristina Delerue-Matos[1]*

[1] REQUIMTE/LAQV, Instituto Superior de Engenharia do Porto, Instituto Politécnico do Porto, rua Dr. António Bernardino de Almeida, 4249-015 Porto, Portugal

[2] REQUIMTE/LAQV, Departamento de Química, Faculdade de Ciências e Tecnologia da Universidade Nova de Lisboa, Quinta da Torre, 2829-516 Caparica, Portugal

[3] Petrogal, S.A., Refinaria de Matosinhos, Rua Belchior Robles, 4451-852 Leça da Palmeira, Portugal

[4] CIQUP, Institute of Molecular Sciences (IMS) - Departamento de Química e Bioquímica, Faculdade de Ciências da Universidade do Porto, Rua do Campo Alegre, P-4169-007 Porto, Portugal

[5]REQUIMTE/LAQV, Departamento de Química e Bioquímica, Faculdade de Ciências,

Universidade do Porto, Rua do Campo Alegre s/n, 4169-007 Porto, Portugal

*corresponding authors: andreia.peixoto@fc.up.pt; elena.surra@graq.isep.ipp.pt; manuela.moreira@graq.isep.ipp.pt;

Number of pages: 26
Number of tables: 4
Number of figures: 15

List of Figures:

Figure S1: a) VPW extract dissolved in benzyl alcohol (BA); b) VPW (1500 ppm) dissolved in BA and added to the biodiesel sample

Figure S2. Typical screen shot view of the software RANCI_Data (right) and RANCI_Cal (left) software applications

Figure S3: Comparation of results of the RANCITECH with a commercial apparatus (Metrohm, Rancimat model 743).

Figure S4: Analysis of VPW extract: a) FTIR spectrum; b) SEM images.

List of Tables:

Table S1: Life Cycle Inventory assumptions made for the design of the sub-processes of Vineyard Pruning Waste (VPW) extract production.

Table S2: Inventory data of the VPW based antioxidant production process. All values are referred to the functional unit (1L biodiesel).
Table S3: Tier 1 Emission Factors for Road transport - Heavy Duty Vehicles[1].

Table S4: Tier 1 emission factors for non-road machinery [2].

Table S5: Inventory data of the landfill of hydrochar. All values are referred to the functional unit (1L biodiesel).

Table S6: Life Cycle Inventory assumptions made for synthetic BHT production process.

Table S7: Inventory data of the BHT production process. All values are referred to the functional unit (1L biodiesel).

Table S8: Chemical composition (Fatty acid methyl ester composition) of the sample biodiesel (antioxidant free) by gas-chromatography (GC) following the EN14103

Table S9: Some properties of the sample biodiesel (antioxidant free)

Table S10: Flash point determination according Procedure C applicable to fatty acid methyl esters (FAME) as specified in EN 14214[11] or ASTM D6751.

Table S11: Processes contribution to the area of protection Human Health at EndPoint(H) level (weighting) of VPW extract dissolved in benzylic alcohol (cut-off 2%). Values expressed as single score (Pt) and percentage (%).

Table S12: Inventory data of the Sensitivity Analysis-1. All values are referred to the functional unit (1L biodiesel).

Table S13: Inventory data of the Sensitivity Analysis-2. All values are referred to the functional unit (1L biodiesel).

Table S14. Total impacts calculated for the different scenarios the Sensitivity Analysis-1 and -2 according to ReCiPe Midpoint (H) method. Light green and yellow cells represent the first and second lowest environmental impacts values. Light orange cells indicate the highest values per each category.

Table S15. Total impacts aggregated in Human Health (DALY), Ecosystems (species.y) and Resource (US$2013) areas of protection according to ReCiPe Endpoint (H) method calculated for the different scenarios the Sensitivity Analysis-1 and -2. Light green and yellow cells represent the first and second lowest environmental impacts values. Light orange cells indicate the highest values per each area of protection.

SI.1 - Materials and Methods : Sample preparation

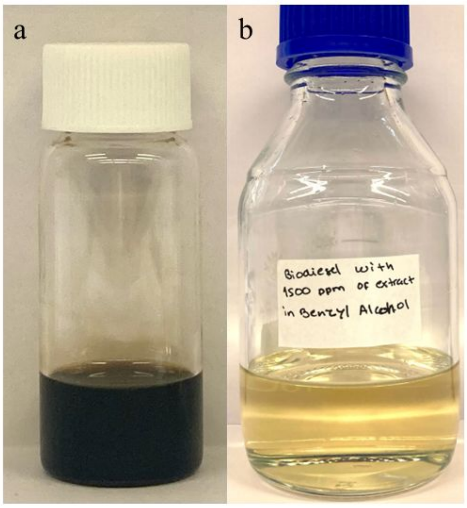

Figure S1: a) VPW extract dissolved in benzyl alcohol (BA); b) VPW (1500 ppm) dissolved in BA and added to the biodiesel sample.

SI.1 - Materials and Methods: RANCItech software

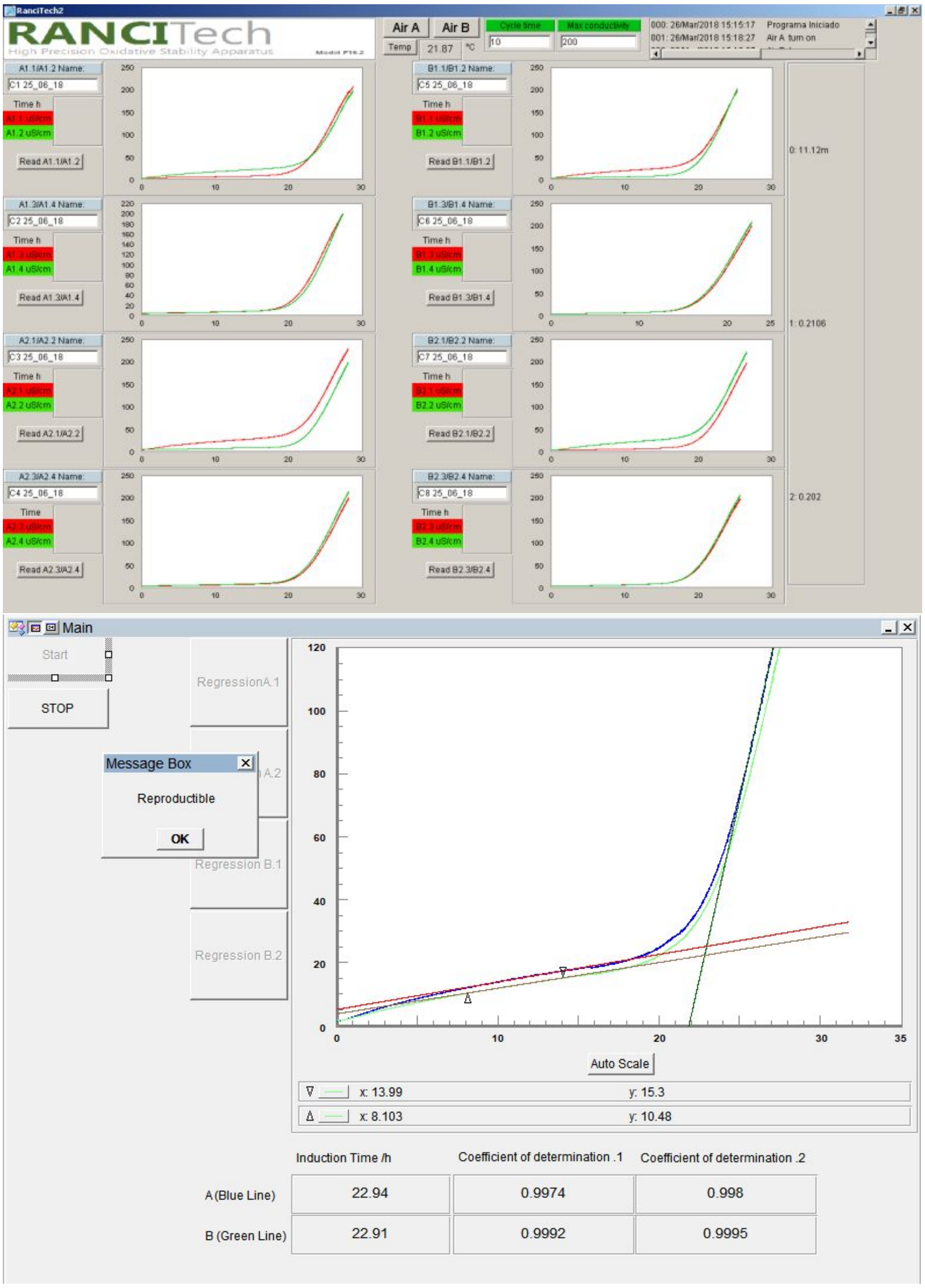

Figure S2: Typical screen shot view of the software RANCI_Data (right) and RANCI_Cal (left) software applications.

SI.1 - Materials and Methods : Comparison of results of the RANCITECH with a commercial apparatus[1]

(Metrohm, Rancimat model 743)

The analysis of performance and accuracy between the IP (induction period) results obtained in the new RANCITECH apparatus was done by comparison of the results obtained for the same sample batch of biodiesel. The obtained data is summarized in Figure S3 which presents a comparison between the results (average of replicas) the two blocks of the Rancimat (Rancimat 1 & Racimat 2) and the RANCITECH results (average of replicas) the four blocks (A1.1, A1.2, B2.1, B2.2). Comparing the results obtained in both studies it was possible to conclude that the RANCITECH can produce reliable values for the OS with a higher reproducibility and lower uncertainty.

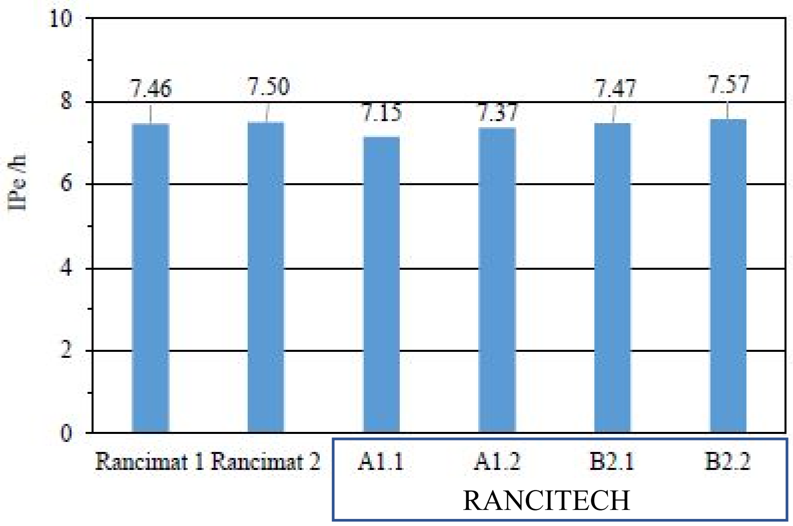

Figure S3: Comparison of results of the RANCITECH with a commercial apparatus (Metrohm, Rancimat model 743).

It was found a higher reproducibility and repeatability of the RANCITECH apparatus which was attributed to the improved air flow control and the better temperature control and homogeneity of the thermostable block.

Based on results obtained in this study, and in the uncertainty propagation analysis (considering the the partial uncertainties of air flow, time interval, temperature oscillation, and sampling) the following uncertainty equation (equation [S1]) was derived for the

[1] Branco, N. F. M. ESTUDO DA ESTABILIDADE OXIDATIVA E DO COMPORTAMENTO A BAIXAS TEMPERATURAS DE MISTURAS DE GASÓLEO COM BIODIESEL, PhD Thesis, 2019. http://hdl.handle.net/10773/26663

estimation of the measuring uncertainty (95 % of confident interval), U of the IP (induction period 7 hours)

$$U\ (IP\ /\ hours) = \pm\ (0.02 \cdot IP\ + 0.1) \qquad \text{(Eq. S1)}$$

The uncertainty results obtained by the derived uncertainty equation (which was derived adopting very conservative uncertainty criterions) are in agreement with the uncertainty results obtained in the test set results and satisfy the BS EN 14112:2020 for the measurement of the induction period (IP).

SI.2 - Results and Discussion

a)

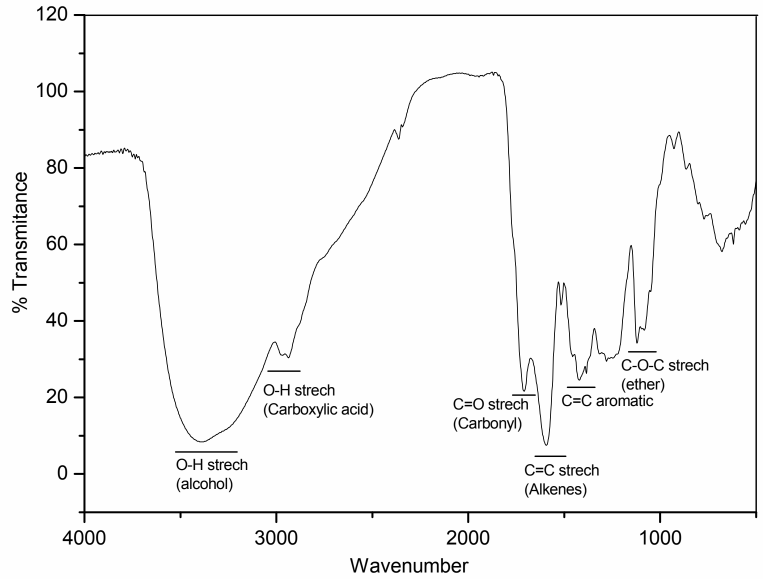

b)

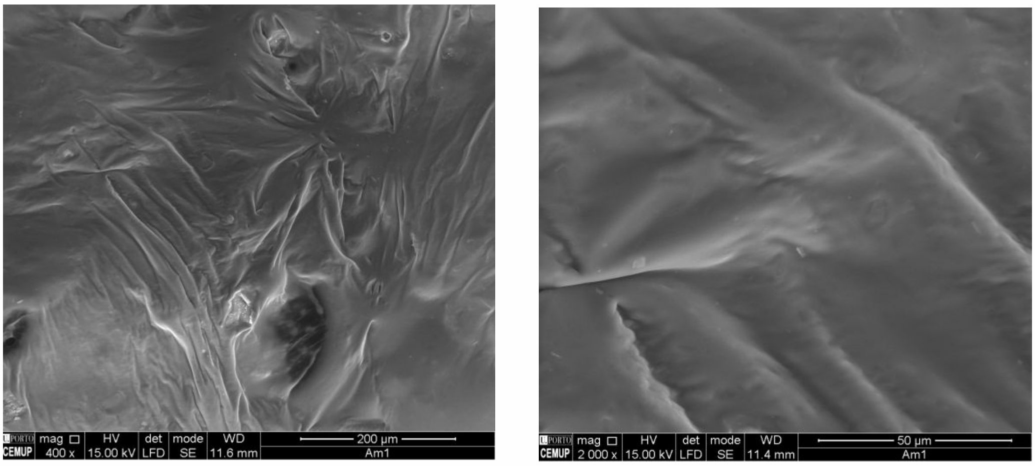

Figure S4: Analysis of VPW extract: a) FTIR spectrum; b) SEM images.

Table S1: Life Cycle Inventory assumptions made for the design of the sub-processes of Vineyard Pruning Waste (VPW) extract production.

**1.VPW Harvest**
The harvest is performed by mechanical box-pruning of 50 ha of "Quinta dos Carvalhais" vineyard (Mangualde, Portugal), assuming the use of a dedicated machinery with average fuel consumption 17.0 L diesel/ha[3]. The harvested vineyard pruning are then collected and transported to the vineyards borders by a support tractor, whose fuel consumption is estimated in 3L diesel/ha [4]. For this calculations, a vineyard pruning density of 720 kg/m^3 with a 50 % w/w moisture content (MC) is considered [5]. Table S2 reports the emission factor assumed for non-road agricultural machineries used for the calculation of emission to air [2]. A consumption of 834.1 kg/y of agricultural diesel is expected. The harvest of vineyard pruning avoided the production of the equivalent amount of wood waste, which avoid consequently the impacts associated to its combustion in a biomass furnace (Table S2) (Waste wood, untreated, {ROW}| heat production, untreated waste wood, at furnace 1000-5000 kW | Consequence, U).
**2. VPW Transport to "Quinta dos Carvalhais"**
The collected VPW are then transported from the vineyard border to "Quinta dos Carvalhais" by a truck with 7 m^3 capacity. Assuming an average distance of 10 km and an estimated fuel consumption of 25 L/100km [6][6], 87.2 kg/y fuel are required. Table S3 reports the emission factor used for the calculation of the emission to air for road transport-heavy duty vehicles [7][7].
**3. VPW Grinding.**
VPW are ground by a wood branches pulverizer, 1000 kg/h, 39.5 kW (53 Hp) capacity able to grind VPW up to 4 mm. The estimate time for pulverizing is 64.5 h/y. As first approach, it is assumed that this particle size allows the same yields of antioxidant activity of 229 mgGAE/g dw considered for the present work [8].
**4. Dried VPW Transport to SWE Plant (Porto, Portugal).**
The ground VPW are transported to SWE plant by Euro VI trucks of 32 m^3 capacity. Assuming an average distance of 150 km, and an estimated fuel consumption of 30 L/100km [6], 674.8 kg/y fuel are required. The emission factor assumed for road transport-heavy duty vehicles used for the calculation of emission to air are reported in Table S3 [7].

Table S1: *cont.*

**5. VPW Feed (Screw-feed).**
For the loading of the ground VPW a standard pitch, single flight a 4 m long, 1000 kg/h capacity, 112 rpm, 0.12 kW screw conveyor was hypothesized. The design of the equipment was performed according to [9], taking sawdust as reference material with an overload and drive efficiency factors of 3 and 0.88, respectively. The water required for the SWE process was 586 m^3/y (ground VPW:water ratio = 10), and it is assumed to be loaded using the grid pressure, so no additional energy requirements are considered for water feed;
**6.Subcritical Water Extraction**
The impact associated to SWE process were calculated based on the enthalpy change required to rise the ground VPW and the water from NTP conditions (20 °C, 1.01325 bar) to 280 °C and 80 bar (SWE operational conditions). The calculation was performed using average specific heat for water ($\overline{cp}$, kJ/kg. °C) calculated between 20 °C and 280 °C according to Eq.S2, $$\overline{cp}_{water} = \frac{\int_{T_1}^{T_2} C_P\,(T)dT}{\int_{T_1}^{T_2} dT} \quad \text{Eq.S2)}$$ which gives 4.73 kJ/kg. °C. In lack of specific experimental data available, the cp for grind VPW was considered constant and equal to 0.9 kJ/kg. °C [10]. The energy required for SWE process was calculated as enthalpy change according to Eq.S3: $$\Delta H = (m_{vine-canes} \times cp_{\,vine-canes} + m_{water} \times \overline{cp}_{\,water}) \; \times (\,T_2 - T_1)\,) \qquad \text{Eq.S3),}$$ where m is the total mass (kg) of ground VPW and water, cp/$\overline{cp}$ are the specific/average specific heat (kJ/kg. °C), respectively, T_2 is the SWE operational temperature (280 °C) and T_1 is the initial room temperature (20 °C). The calculated heat amount required for SWE according to [11], is 235.7 MWh/y.
**7. Flash and Separation of hydrochar from liquid VPW extract**
To allow heat recover and energy saving, 174 MWh of heat in form of water vapor are recovered hypothesizing the sudden pressure release from SWE reactor from 80 bar to almost atmospheric pressure (1.1 bar). This flash allows to vaporize the hot liquid water present in the SWE reactor, recovering a hot vapor stream and reducing the remaining water vapor that must be evaporated at the end by 46 % w/w. Based on the phenolic composition of the VPW extract, which includes (+)- catechin acid (24% w/w), gallic acid (16%w/w), (-)- epicatechin acid (14% w/w), caffeic acid (9% w/w), chlorogenic acid (7% w/w), others (30% w/w) [8], it is assumed that no valuable antioxidants are drag into the

exiting steam stream, since all of them have boiling point higher than 400 °C.

Table S1: *cont.*

**7. Flash and Separation of hydrochar from liquid VPW extract**
Thus, the remaining concentrated-SWE extract is separated from the hydrochar by filtering in the presence of a pump of 4 bar discharge pressure, which demands 0.034 MWh/y electric energy. Two different pathways were considered for the hydrochar separated from concentrated liquid extract, namely: (i) hydrochar transport and landfill, assuming 20 km distance from the landfill and the use of a diesel fueled truck for hydrochar transportation (Table S5), and the (ii) introduction into the market as precursor for AC. This second option allows the "avoided production" of an equivalent amount of charcoal, providing environmental credits to the system, since the "system expansion" approach was adopted (Figure 2a). For the modeling of these two scenarios, they have been used the reference processes included in the Ecoinvent 3.7 database "Wood ash mixture, pure {Europe without Switzerland} | treatment of wood ash mixture, pure, sanitary landfill| Conseq, U") (Table S4) and "Charcoal {GLO} | market for | Conseq, U", respectively (Table S2);
**8. Concentrated VPW extract Evaporation**
The remaining condensate SWE extract is evaporated in a thin film evaporator, operated in continuous under vacuum (0.01 bar) in the presence of a chiller used to lower the temperature of the incoming concentrated VPW extract to 33 ℃. The refrigeration of outcoming stream from SWE reactor and the generation of vacuum requires additional 193.28 MWh/y electric energy, with the chiller representing more than 99% of this demand.<br>The vacuum has the function of lowering the boiling point of water from 100 ℃ to 9.7 ℃ and to achieve then thermodynamic conditions which allow the recovery of the 174 MWh/y of heat produced during the water vapor flash (sub-process 7) for final evaporation. The design of the energy integration in the evaporation process allows to avoid the production of an equivalent amount of heat, based on the process "Heat, central or small scale, natural gas {Europe without Switzerland}, market for |Conseq, U" of Ecoinvent 3.7 database, since the heat required for the evaporation of the remaining concentrated SWE extract accounts is almost equivalent to the heat recovered from the flash. The condensate is then collected and sent to treatment according to the process included in Ecoinvent 3.7: "Wastewater, average {Europe without Switzerland} | treatment of wastewater, average, capacity 1E9l/year | Conseq, U");
**9. Dried VPW extract Dissolution in benzyl alcohol**
According to the laboratorial conditions tested, which were properly scaled-up, the use of 697.8 t/y of benzyl alcohol (ratio 0.02 $kg_{extract}$:$L_{alcohol\ benzyl}$), was considered to dissolve the precipitated antioxidant extract and to obtain the final usable biodiesel additive.

.

Table S2: Inventory data of the VPW based antioxidant production process. All values are referred to the functional unit (1L biodiesel).

Sub-Process	Unit	Value
**1.VPW Harvest**		
INPUT		
VPW	kg/L	7.21E-03
Diesel {Europe without Switzerland}, market for|Conseq, U	kg/L	1.12E-04
OUTPUT		
Harvested VPW	kg/L	7.21E-03
Emission to air		
CO	kg/L	1.23E-06
CO_2	kg/L	3.54E-04
N_2O	kg/L	1.52E-08
NOx	kg/L	3.92E-06
NMVOC	kg/L	3.77E-07
NH_3	kg/L	8.96E-10
CH_4	kg/L	6.16E-09
PM_{10}	kg/L	1.95E-07
$PM_{2.5}$	kg/L	1.95E-07
**2.VPW Transport to "Quinta do Carvalhais”**		
INPUT		
Harvested VPW	kg/L	7.21E-03
Diesel, low sulphur {Europe without Switzerland}, market for|Conseq, U	kg/L	9.96E-06
OUTPUT		
VPW transported to "Quinta do Carvalhais"	kg/L	7.21E-03
Emission to air		
CO	kg/L	7.55E-08
CO_2	kg/L	3.13E-05
N_2O	kg/L	5.08E-10
NOx	kg/L	3.32E-07
NMVOC	kg/L	1.19E-08
NH_3	kg/L	1.29E-10
PM	kg/L	1.99E-10
Waste and emission to treatment		
Waste wood, untreated, {ROW}| heat production, untreated waste wood, at furnace 1000-5000 kW | Consequence, U	kg/L	-9.83E-03

Table S2: *cont.*

Sub-Process	Unit	Value
**3.VPW Griding**		
INPUT		
VPW transported to "Quinta do Carvalhais"	Kg/L	7.21E-03
Electricity, low voltage {PT}, market for|Conseq, U	kWh/L	2.85E-04
OUTPUT		
Ground VPW	kg/L	6.55E-03
Waste		
Sawdust, loose, wet, measured as dry mass {GLO}, market for|Conseq, U	kg/L	6.55E-04
**4.VPW transport to SWE Plant (Porto)**		
INPUT		
Ground VPW	kg/L	6.55E-03
Diesel, low sulphur {Europe without Switzerland}, market for|Conseq, U	kg/L	7.54E-05
OUTPUT		
VPW transported to SWE Plant (Porto)	kg/L	6.55E-03
Emission to air		
CO	kg/L	5.72E-07
CO_2	kg/L	2.37E-04
N_2O	kg/L	3.85E-09
NOx	kg/L	2.52E-06
NMVOC	kg/L	1.45E-07
NH_3	kg/L	9.80E-10
PM	kg/L	3.02E-08
**5.VPW Feed (Screw-feeder)**		
INPUT		
VPW transported to SWE Plant (Porto)	kg/L	6.55E-03
Electricity, low voltage {PT}, market for|Conseq, U	kWh/L	7.86E-07
OUTPUT		
VPW (Screw fed)	kg/L	6.55E-03

Table S2: *cont.*

Sub-Process	Unit	Value
**6.Subcritical Water Extraction**		
INPUT		
VPW (Screw fed)		6.55E-03
Heat, central or small scale, natural gas {Europe without Switzerland}, market for |Conseq, U	kWh/L	2.63E-02
Tap water {Europe without Switzerland}, market for|Conseq, U	kg/L	6.55E-02
Electricity,low voltage {PT}, market for|Conseq, U	kg/L	3.80E-06
OUTPUT		
SWE-liquid Extract	kg/L	6.51E-02
Hydrochar	kg/L	6.92E-03
**7.Flash Liquid- Vapor**		
INPUT		
SWE-liquid Extract	kg/L	6.51E-02
Hydrochar	kg/L	6.92E-03
OUTPUT		
Concentrated SWE Extract	kg/L	3.46E-02
Heat from flash	kWh/L	1.94E-02
Water Vapor	kg/L	3.05E-2
Hydrochar	kg/L	6.92E-03
Scenary: Hydrochar Landfill: Ref. Table S5		
Scenary: Hydrochar as precursor for AC		
Avoided Product		
Charcoal {GLO}, market for |Conseq, U	kg/L	6.92E-03
**8.VPW Extract Precipitation**		
INPUT		
Concentrated SWE Extract	kg/L	3.46E-02
Water Vapor	kg/L	3.05E-2
Electricity,low voltage {PT}, market for|Conseq, U	kWh	2.16E-2
OUTPUT		
VPW extract precipitated	kg/L	1.50E-03
Avoided Product		
Heat, central or small scale, natural gas {Europe without Switzerland}, market for |Conseq, U	kg/L	1.94E-02
Emission to water		
Condensed water	kg/L	6.36E-02

Table S2: *cont.*

**Sub-Process**	**Unit**	**Value**
**9.Dissolution in benzyl alcohol**		
INPUT		
VPW extract precipitated	kg/L	1.50E-03
Benzyl Acohol {GLO} | market for | Conseq, U	kg/L	7.80E-02
Electricity,low voltage {PT}, market for|Conseq, U	kWh	2.16E-02
OUTPUT		
Dissolved VPW extract	kg/L	7.95E-02

Table S3: Tier 1 Emission Factors for Road transport - Heavy Duty Vehicles [1]

**Gas**	**Unit**	**Value**
CO	kg/kg diesel	7.6E-03
CO_2		3.1E+00
N_2O		5.1E-05
NOx		3.3E-02
NMVOC		1.9E-03
NH_3		1.3E-05
PM		2.0E-05

Table S4: Tier 1 emission factors for non-road machinery [2]

**Gas**	**Unit**	**Value**
CO	kg/kg diesel	1.09E-02
CO_2		3.16E+00
N_2O		1.36E-04
NOx		3.50E-02
COVNM		3.37E-03
NH_3		8.00E-06
CH_4		5.50E-05
PM10		1.74E-03
PM2.5		1.74E-03

Table S5: Inventory data of the landfill of hydrochar. All values are referred to the functional unit (1L biodiesel).

Sub-Process	Unit	Value
**Hydrochar transport to landfill**		
INPUT		
Hydrochar	kg/L	6.92E-03
Diesel {Europe without Switzerland}, market for|Conseq, U	kg/L	4.15E-02
OUTPUT		
Hydrochar transported to landfill	kg/L	7.21E-03
Emission to air		
CO	kg/L	3.15E-04
CO_2	kg/L	1.30E-01
N_2O	kg/L	2.12E-06
NOx	kg/L	1.39E-03
NMVOC	kg/L	7.98E-05
NH_3	kg/L	5.40E-07
PM_{10}	kg/L	0.83E-06
$PM_{2.5}$	kg/L	0.83E-06
**Hydrochar Landfill**		
INPUT		
Hydrochar transported to landfill (by	kg/L	6.92E-03
Wood ash mixture, pure {Europe without Switzerland} | treatment of wood ash mixture, pure, sanitary landfill| Conseq, U)	kg/L	2.63E-02
OUTPUT		
Hydrochar landfilled	kg/L	6.92E-03

Table S6: Life Cycle Inventory assumptions made for the design of the synthetic BHT production process.

**BHT synthesis**
The LCI of the production process of BHT assumes that oxidative stability of biodiesel is achieved with 600 ppm of BHT addition and that 5,368 kg/y of BHT are required to equal the correspondent amount of VPW extract produced (Table S7). As first approximation, in absence of specific data in Ecoinvent 3.7 database, the estimate of the environmental impacts associated with the BHT production process was based on the data retrieved by the US Patent 2,428,745 [12] integrated with the experimental results obtained by Yadav et al [13], where the synthetic antioxidant BHT is obtained by alkylation of p-cresol with isobutylene in the presence of sulfated zirconia (S-ZrO_2) as superacid catalyst. The isobutylene amount required was assumed, as first approximation, 100% excess w/w of the amount calculated through the mass balance of the experimental results obtained by Yudav et al. [13]. Alkylation of p-cresol with isobutylene is a combination of series and parallel steps proceeding via an intermediate. The reaction products are the monoalkylated and dialkylated p-cresols and the oligomerized isobutylene leading to dimer, trimer, and tetramer: 9.6x10^3 g.mol.cm^{-3} p-cresol gives 1.4x10^3g.mol.cm^{-3} 2-tert-butyl-p-cresol; 7.5x10^3 g.mol.cm^{-3}; 0.6 $\times$ 10^3 g.mol.cm^{-3} diisobutylene, 1.4 g.mol/cm^{-3} triisobutylene, 0.6 g.mol.cm^{-3} tetraisobutylene in the presence of 3% (w/w) S-ZrO_2 at 348 K [13].
**p-Cresol synthesis**
The p-cresol production process was modeled assumed a two steps route based on the (i) sulfonation of toluene and successive (ii) basic hydrolysis of the sulphonate salt, which gives p-cresol and sodium sulphite according to Eq. S4 and S5:<br><br>$CH_3C_6H_5 + H_2SO_4 \rightarrow CH_3C_6H_4SO_3H + H_2O$ Eq. S4)<br><br>$CH_3C_6H_4SO_3H + 2NaOH \rightarrow CH_3C_6H_4OH + Na_3SO_3 + H_2O$ Eq. S5)<br><br>The indirect impacts associated to toluene, sulphuric acid, sodium hydroxide, and isobutylene production processes were calculated based on the Ecoinvent 3.7 database: “toluene, liquid {RER} |, production| Conseq, U”, “sulfuric acid, at plant/kg/, sodium hydroxide, production mix, at plant/RNA, “Butene, mixed {RER} |, production| Conseq, U”.

Table S6: *cont.*

**Catalyst production**
According to the methodology suggested by Yadav et al. [13] the catalyst used for the p-cresol synthesis is sulfated zirconia (S-ZrO_2 or $Zr(SO_4)_2$). In the absence of a specific process in Ecoinvent 3.7 database, the assessment of the indirect environmental impacts associated to the production of this catalyst, it was created an *ad hoc* process, based on the following assumption:<br>• zirconium dioxide (ZrO_2) is used as precursor for sulfated zirconia synthesis, since its industrial production process can be conservatory considered equal to that of zirconium oxychloride ($ZrOCl_2 \cdot 8H_2O$) [14], which is referred to be raw material for catalyst production. The indirect impacts associated to ZrO_2 production processes were calculated based on the Ecoinvent 3.7 database: "Zircon, 50%, zirconium, {GLO} |, market for| Conseq, U". Proper quantity adjustments were made to guarantee the correct amount of ZrO_2;<br>• the ammonia induced hydroxide precipitation from zirconium oxychloride was considered to be as in Eq. S6 [15]:<br>$ZrO^{+2} + 2Cl^{-} + 2NH_4OH \Leftrightarrow ZrO(OH)_2 + 2NH_4Cl$ (Eq. S6)<br>• the impregnation of zirconium hydroxide was performed by immersing the dried zirconium hydroxide in 0.5M H_2SO_4 in the ratio of 1:30 w/w;<br>• the energy required for S groups calcination was calculated by enthalpy change from 25 ℃ to 650 ℃, considering the constant pressure heat capacity of sulfuric acid of 1.380 J.(kg.K)$^{-1}$ [16], of water of 4.190 J.(kg.K)$^{-1}$ [16] and of zirconium of 270 J.(mol.K)$^{-1}$ [16], which gives 2.293 kWh/y.
**Waste**
The amount produced of diisobutylene, triisobutylene, tetraisobutylene and 2-tert-butyl-p-cresol are considered as hazardous liquid waste exiting in the system.
**Energy**
The energy required for reaching the reaction temperature was calculate as enthalpy change from 298 K to 348 K, considering the constant pressure heat capacity of p-cresol of 163 J/mol.K [16] and of isobutylene of 88.09 J/mol.K [16] applying the Eq. S7.<br>$\Delta H = (m_{p-cresol} \times cp_{p-cresol} + m_{isobuthylene} \times cp_{isobuthylene}) \times \Delta\,(T_2 - T_1)$ (Eq. S7)<br>It accounts for 350 kWh/y. The final raw BHT obtained is washed with a NaOH 5% w/v solution, whose yield is considered to be 46% [12].

Table S7: Inventory data of the BHT production process. All values are referred to the functional unit (1L biodiesel).

Subprocess	Unit	Value
**p-cresol production - STEP 1**		
INPUT		
Toluene, liquid {RER} |, production| Conseq, U	kg/L	6.98E-04
Sulphuric acid, at plant/kg/RNA		7.43E-04
OUTPUT		
p-toluene sulfonic acid		1.36E-04
Waste and Emissions to treatment		
Wastewater, average {Europe without Switzerland} | treatment of wastewater, average, capacity 1E9l/year | Conseq, U		1.30E-03
**p-cresol production - STEP 2**		
INPUT		
p-toluene sulfonic acid	kg/L	1.30E-03
Sodium Hydroxide, production mix, at plant/RNA		6.06E-04
OUTPUT		
p-cresol		8.19E-04
Waste and Emissions to treatment		
Wastewater, average {Europe without Switzerland} | treatment of wastewater, average, capacity 1E9l/year | Conseq, U		1.36E-04
Sodium sulphate (wfr) /RER		9.55E-04
**Raw BHT Production**	Unit	Value
INPUT		
p-cresol	kg/L	8.19E-04
Butene, mixed {RER} |, production| Conseq, U		1.01E-03
Heat, district or industrial, natural gas {RER} |, market group for| Conseq, U	kwh/L	1.72E-05
OUTPUT		
Raw BHT	kg/L	1.30E-03
Waste and Emissions to treatment		
Dummy_Disposal, chemcal waste, unspecified, to residual material landfill/kg/GLO		5.27E-04

Table S7: *cont.*

Subprocess	Unit	Value
**Raw BHT Washing**		
INPUT		
Raw BHT		1.30E-03
Sodium Hydroxide, production mix, at plant/RNA		6.52E-05
Tap water {Europe without Switzerland} |, tap water production, underground water without treatment | Conseq, U		1.24E-03
OUTPUT	kg/L	
BHT crystals		6.00E-04
Waste and Emissions to treatment		
Wastewater, average {Europe without Switzerland} | treatment of wastewater, average, capacity 1E9l/year | Conseq, U		2.01E-03

Table S8: Chemical composition (Fatty acid methyl ester composition) of the sample biodiesel (antioxidant free) by gas-chromatography (GC) following the EN14103.

Fatty acid methyl	Biodiesel/without antioxidant	
	Concentration (% m/m)	Method/Norm
C8:0	0.40	EN14103
C10:0	0.33	EN14103
C12:0	2.22	EN14103
C14:0	1.70	EN14103
C15:1	0.34	EN14103
C16:0	27.99	EN14103
C16:1	0.75	EN14103
C17:0	0.18	EN14103
C17:1	0.11	EN14103
C18:0	5.65	EN14103
C18:1	38.23	EN14103
C18:2	16.50	EN14103
C18:3	0.57	EN14103
C20:0	0.38	EN14103
C21:0	0.21	EN14103
Others	3.03	EN14103

Table S9: Some properties of the sample biodiesel (antioxidant free)

**Property**	Biodiesel/without antioxidant	
	Value	Method/Norm
Linolenic Acid methyl ester (% m/m)	0.57	EN14103
Water Content Karl-Fischer (% m/m)	0.010	ISO 12937
Esters content (% m/m)	98.58	EN14103
Density at 15 ºC (g/ml)	0.8766	ASTM D4052
Flash point (ºC)	160	EN14103
Cloud point (ºC)	8	ASTM D2500
Acidity (mg KOH /g)	0.32	EN14104
Iodine Value (g /100 g)	77	EN14111

Table S10: Flash point determination using ISO 2719 - Procedure C (applicable to fatty acid methyl esters (FAME).

Sample name	Flash point, ˚C
BD	158.0
BD-BHT 600	128.0
BD-VPW 1500	134.0
BD-BA	126.0

Table S11: Processes contribution to the area of protection Human Health at EndPoint(H) level (weighting) of VPW extract dissolved in benzylic alcohol (cut-off 2%). Values expressed as single score (Pt) and percentage (%).

Process	Hydrochar as AC precursor		Hydrochar Landfill	
	kPt	%	kPt	%
Total of all processes	135.1462	100.0	135.2646	100.0
Remaining processes	63.0849	46.7	63.1984	46.7
Toluene, liquid {RoW}| production | Conseq, U	19.4665	14.4	19.4665	14.4
Heat, district or industrial, other than natural gas {RoW}| heat and power co-generation, lignite | Conseq, U	18.4094	13.6	18.4099	13.6
Electricity, high voltage {RoW}| electricity production, lignite | Conseq, U	13.5390	10.0	13.5425	10.0
Spoil from lignite mining {GLO}| treatment of, in surface landfill | Conseq, U	5.6154	4.2	5.6162	4.2
Heat, district or industrial, other than natural gas {RU}| heat and power co-generation, lignite | Conseq, U	5.3569	4.0	5.3570	4.0
Toluene, liquid {RER}| production | Conseq, U	4.1007	3.0	4.1007	3.0
Heat, district or industrial, natural gas {RoW}| heat and power co-generation, natural gas, 200kW electrical, lean burn | Conseq, U	3.5758	2.6	3.5759	2.6
Copper {RAS}| production, primary | Conseq, U	3.0225	2.2	3.0227	2.2
Heat, district or industrial, other than natural gas {RU}| heat and power co-generation, hard coal | Conseq, U	2.8983	2.1	2.8984	2.1
Palladium {RU}| platinum group metal mine operation, ore with high content | Conseq, U	-3.9231	-2.9	-3.9235	-2.9

Table S12: Inventory data of the Sensitivity Analysis-1. All values are referred to the functional unit (1L biodiesel).

**Scenario "VPW in Pentanol"**	**Unit**	**Value**
INPUT		
VPW extract precipitated	kg/L	1.50E-03
1-pentanol {GLO} | market for | Conseq, U [1)]	kg/L	6.08E-02
Electricity,low voltage {PT}, market for|Conseq, U	kWh	2.16E-02
OUTPUT		
Dissolved VPW extract	kg/L	6.23E-02

**Scenario "VPW in Ethylene Glycol"**	**Unit**	**Value**
INPUT		
VPW extract precipitated	kg/L	1.50E-03
Ethylene glycol |{RER} | production | Conseq, U[2)]	kg/L	8.33E-02
Electricity,low voltage {PT}, market for|Conseq, U	kWh	2.16E-02
OUTPUT		
Dissolved VPW extract	kg/L	8.48E-02

[1)] 1-pentanol density: 0.811 g/cm^3 (25ºC)
[2)] ethylene glycol density: 1.11 g/cm^3 (25ºC)

Table S13: Inventory data of the Sensitivity Analysis-2. All values are referred to the functional unit (1L biodiesel).

**Scenario "VPW in BA 25"**	**Unit**	**Value**
INPUT		
VPW extract precipitated	kg/L	1.50E-03
Benzyl Acohol {GLO} | market for | Conseq, U	kg/L	6.24E-02
Electricity,low voltage {PT}, market for|Conseq, U	kWh	2.16E-02
OUTPUT		
Dissolved VPW extract	kg/L	6.39E-02

**Scenario "VPW in BA 40"**	**Unit**	**Value**
INPUT		
VPW extract precipitated	kg/L	1.50E-03
Benzyl Acohol {GLO} | market for | Conseq, U	kg/L	3.90E-02
Electricity,low voltage {PT}, market for|Conseq, U	kWh	2.16E-02
OUTPUT		

Dissolved VPW extract	kg/L	4.05E-02

Table S14. Total impacts calculated for the different scenarios the Sensitivity Analysis-1 and -2 according to ReCiPe Midpoint (H) method. Light green and yellow cells represent the first and second lowest environmental impacts values. Light orange cells indicate the highest values per each category.

				Sensitivity Analysis 1		Sensitivity Analysis 2	
Impact category	Unit	Commercial BHT	VPW in BA	VPW in Pentanol	VPW in Ethylene Glycol	VP in BA 25	VPW in BA 40
Global Warming	kg CO_2 (eq.)	1.49×10^{4}	3.35×10^{6}	4.35×10^{6}	1.02×10^{6}	3.32×10^{6}	3.25×10^{6}
Stratospheric ozone depletion	kg CFC11 (eq.)	2.66×10^{-2}	1.73	9.79×10^{-1}	2.29×10^{-1}	1.72	1.69
Ionizing radiation	kBq Co-60 (eq.)	3.37×10^{2}	3.51×10^{4}	-2.63×10^{5}	1.12×10^{5}	3.51×10^{4}	3.51×10^{4}
Ozone formation, Human health	kg NOx (eq.)	4.28×10^{1}	6.14×10^{3}	8.69×10^{3}	1.93×10^{3}	6.11×10^{3}	6.03×10^{3}
Fine particulate matter formation	kg $PM_{2.5}$ (eq.)	3.22×10^{1}	5.55×10^{3}	1.12×10^{4}	1.27×10^{3}	5.53×10^{3}	5.46×10^{3}
Ozone formation, Terrestrial ecosystems	kg NOx (eq.)	4.43×10^{1}	6.48×10^{3}	9.03×10^{3}	2.08×10^{3}	6.45×10^{3}	6.36×10^{3}
Terrestrial acidification	kg SO_2 (eq.)	8.26×10^{1}	7.35×10^{3}	1.55×10^{4}	2.55×10^{3}	7.33×10^{3}	7.26×10^{3}
Freshwater eutrophication	kg P (eq.)	2.00×10^{1}	1.41×10^{3}	3.43×10^{3}	3.63×10^{2}	1.40×10^{3}	1.39×10^{3}
Marine eutrophication	kg N (eq.)	1.05×10^{2}	1.18×10^{2}	2.07×10^{2}	2.14×10^{1}	1.18×10^{2}	1.16×10^{2}
Terrestrial ecotoxicity	kg 1.4-DCB	6.61×10^{4}	1.21×10^{7}	4.29×10^{6}	5.20×10^{6}	1.21×10^{7}	1.23×10^{7}
Freshwater ecotoxicity	kg 1.4-DCB	1.13×10^{3}	2.40×10^{5}	8.64×10^{4}	1.35×10^{5}	2.42×10^{5}	2.48×10^{5}
Marine ecotoxicity	kg 1.4-DCB	1.47×10^{3}	3.05×10^{5}	1.25×10^{5}	1.70×10^{5}	3.08×10^{4}	3.15×10^{5}
Human carcinogenic toxicity	kg 1.4-DCB	1.52×10^{3}	1.06×10^{5}	1.90×10^{5}	3.51×10^{4}	1.06×10^{4}	1.06×10^{5}
Human non-carcinogenic toxicity	kg 1.4-DCB	5.66×10^{4}	4.10×10^{6}	4.81×10^{6}	1.59×10^{6}	4.09×10^{6}	4.09×10^{6}
Land use	m^2a crop (eq.)	6.03×10^{2}	1.36×10^{5}	5.95×10^{5}	6.66×10^{4}	1.35×10^{5}	1.29×10^{5}
Mineral resource scarcity	kg Cu (eq.)	2.21×10^{2}	1.21×10^{4}	2.74×10^{3}	5.79×10^{3}	1.21×10^{4}	1.21×10^{4}

Fossil resource scarcity	kg oil (eq.)	5.97 x10^3	1.46 x10^6	1.81 x10^6	6.70 x10^5	1.45 x10^6	1.42 x10^6
Water consumption	m^3	-1.60 x10^4	7.28 x10^4	1.85 x10^5	2.40 x10^4	7.26 x10^4	7.20 x10^4

BA: Benzyl alcohol; 1.4-DCB: Diclorobenzene

Table S15. Total impacts aggregated in Human Health (DALY), Ecosystems (species.y) and Resource (US$2013) areas of protection according to ReCiPe Endpoint (H) method calculated for the different scenarios the Sensitivity Analysis-1 and -2. Light green and yellow cells represent the first and second lowest environmental impacts values. Light orange cells indicate the highest values per each area of protection.

				Sensitivity Analysis 1		Sensitivity Analysis 2	
Damage category	Unit	Commercial BHT	VPW in BA	VPW in Pentanol	VPW in Ethylene Glycol	VPW in BA 25	VPW in BA 40
Human health	DALY	1.63×10^{-2}	8.03	13.1	2.28	7.99	7.87
Ecosystems	species. y	-1.31×10^{-4}	1.51×10^{-2}	2.62×10^{-2}	4.98×10^{-3}	1.50×10^{-2}	1.47×10^{-2}
Resources	US$2013	2.19×10^{3}	5.13×10^{5}	5.49×10^{5}	2.67×10^{5}	5.10×10^{5}	5.00×10^{5}

References

(1) Ntziachristos, L.; Samaras, Z. *EMEP/EEA air pollutant emission inventory guidebook 2016* (Updated Jul.2018); EEA Report No 21/2016, European Environment Agency, 2016. https://www.eea.europa.eu/publications/emep-eea-guidebook-2016 (accessed 2022-03-15)

(2) Winther, M.; Samaras, Z.; Zierock, K.-H.; Lambrecht, U. *1.A.4 Non-road mobile sources and machinery*, EMEP/EEA Emiss. Invent. Guideb. 2013, European Environment Agency, 2013.

https://www.eea.europa.eu/publications/emep-eea-guidebook-2013/part-b-sectoral-guidance-chapters/1-energy/1-a-combustion/1-a-4-non-road-mobile-sources/view (accessed 2022-03-15)

(3) Kurtural, S. K.; Fidelibus, M. W. Mechanization of Pruning, Canopy Management, and Harvest in Winegrape Vineyards. *Catal. Discov. into Pract.*, **2021**, *5*(1), 29–44. **DOI:** 10.5344/catalyst.2021.20011

(4) Stajnko, D.; Lakota, M.; Vindiš, P. Productivity and fuel consumption of tractor driven inter-row mulcher Lotti IT 26 A in a different slope level vineyards. In *Proceedings of the 45th International Symposium on Agricultural Engineering, Actual Tasks on Agricultural Engineering*, Opatija, Croatia, February 2017, pp. 165–172.

https://www.cabdirect.org/cabdirect/abstract/20173126973

(5) Nasser, R. A.; Salem, M. Z. M.; Al-Mefarrej, H. A.; Abdel-Aal, M. A.; Soliman, S. S. Fuel characteristics of vine prunings (Vitis vinifera L.) as a potential source for energy production. *BioResources*, **2014**, *9*(1), 482–496.

(6) Rodríguez, F.; Delgado, O.; Muncrief, R. *Fuel consumption testing of tractor-trailers in the European Union and the United States,* International Council on Clean Transportation, 2018.

https://theicct.org/publication/fuel-consumption-testing-of-tractor-trailers-in-the-european-union-and-the-united-states/

(7) Ntziachristos, L.; Samaras, Z. *Passenger cars, light commercial trucks, heavy-duty vehicles including buses and motorcycles*, EMEP/EEA air pollutant emission inventory guidebook 2019, European Environment Agency, 2021.

https://www.eea.europa.eu/publications/emep-eea-guidebook-2019/part-b-sectoral-guidance-chapters/1-energy/1-a-combustion/1-a-3-b-i (accessed 2022-03-11)

(8) Moreira, M. M.; Dorosh, O.; Silva, S.; Silva, A. M.; Grosso, C.; Vieira, E. F.; Rodrigues, F.; Fernandes, V. C.; Peixoto, A. F.; Freire, C.; Delerue-Matos., C. Subcritical Water Extraction of Phenolic Compounds from Vineyard Pruning Residues: Evaluation of Chemical Composition and Bioactive Properties. In *Biol. Life Sci. Forum* **2021**, Proceedings of the 2nd International Electronic Conference on Foods - "Future Foods and Food Technologies for a Sustainable World", Electronic Conference, October 2021. DOI: 10.3390/Foods2021-11103

(9) Conveyors, K. Screw Conveyor Engineering Guide 2022. http://www.kaseconveyors.com/bulk-material-handling-products/engineering-guide/conveyor-flight-pitch-types.htm (accessed 2022-03-15)

(10) The Engineering Toolbox, "Solid Specific Heat," 2022. https://www.engineeringtoolbox.com/specific-heat-solids-d_154.html.

(accessed 2022-03-29).

(11) Aspentech. AspenPlus software package.

https://www.aspentech.com/en/academic-program/aspenone-for-academics-products

(accessed 2021-03-01)

(12) Stillson, G. Alkylation of phenols. US2428745A, 1947.

(13) Yadav, G. D.; Thorat, T. S. Kinetics of alkylation of p-cresol with isobutylene catalyzed by sulfated zirconia. *Ind. Eng. Chem. Res.*, **1996**, 35(3), 721–731. DOI: 10.1021/ie940340r

(14) Sajima, H. P.; Pusporini, N. D. Synthesis of Zirconium Oxychloride and Zirconia Low TENORM by Zircon Sand from Landak West Kalimantan. In *J. Phys. Conf. Ser.*, **2020**, Proceeding of International Conference on Nuclear Capacity Building, Education, Research and Applications (I-Concern19), Yogyakarta, Indonesia, September 2019. DOI: 10.1088/1742-6596/1436/1/012106

(15) Carter, G. A.; Ogden, M. I.; Buckley, C. E.; Maitland, C.; Paskevicius, M. Ammonia-induced precipitation of zirconyl chloride and zirconyl-yttrium chloride solutions under industrially relevant conditions. *Powder Technol.*, **2009**, *188*(3), 222–228. DOI: 10.1016/j.powtec.2008.04.087

(16) p-cresol, *NIST Chemistry WebBook, SRD 69*. https://webbook.nist.gov/cgi/cbook.cgi?ID=C106445&Mask=E

(accessed: 2020-03-31)
